# Crystal structure of parallel G-quadruplex formed by the two-repeat ALS- and FTD-related GGGGCC sequence

**DOI:** 10.1093/nar/gkab302

**Published:** 2021-05-28

**Authors:** Yanyan Geng, Changdong Liu, Qixu Cai, Zhipu Luo, Haitao Miao, Xiao Shi, Naining Xu, Chun Po Fung, To To Choy, Bing Yan, Ning Li, Peiyuan Qian, Bo Zhou, Guang Zhu

**Affiliations:** Division of Life Science, The Hong Kong University of Science and Technology, Clear Water Bay, Kowloon, Hong Kong SAR, 00000, China; Division of Life Science, The Hong Kong University of Science and Technology, Clear Water Bay, Kowloon, Hong Kong SAR, 00000, China; Division of Life Science, The Hong Kong University of Science and Technology, Clear Water Bay, Kowloon, Hong Kong SAR, 00000, China; Institute of Molecular Enzymology, School of Biology and Basic Medical Sciences, Soochow University, Suzhou, Jiangsu, 215123, China; Division of Life Science, The Hong Kong University of Science and Technology, Clear Water Bay, Kowloon, Hong Kong SAR, 00000, China; Division of Life Science, The Hong Kong University of Science and Technology, Clear Water Bay, Kowloon, Hong Kong SAR, 00000, China; Division of Life Science, The Hong Kong University of Science and Technology, Clear Water Bay, Kowloon, Hong Kong SAR, 00000, China; Division of Life Science, The Hong Kong University of Science and Technology, Clear Water Bay, Kowloon, Hong Kong SAR, 00000, China; Division of Life Science, The Hong Kong University of Science and Technology, Clear Water Bay, Kowloon, Hong Kong SAR, 00000, China; Division of Life Science, The Hong Kong University of Science and Technology, Clear Water Bay, Kowloon, Hong Kong SAR, 00000, China; Division of Life Science, The Hong Kong University of Science and Technology, Clear Water Bay, Kowloon, Hong Kong SAR, 00000, China; Hong Kong Branch of Southern Marine Science and Engineering Guangdong Laboratory (Guangzhou), Hong Kong University of Science and Technology, Clear Water Bay, Kowloon, Hong Kong SAR, 00000, China; Division of Life Science, The Hong Kong University of Science and Technology, Clear Water Bay, Kowloon, Hong Kong SAR, 00000, China; Institute for Advanced Study, The Hong Kong University of Science and Technology, Clear Water Bay, Kowloon, Hong Kong SAR, 00000, China; Division of Life Science, The Hong Kong University of Science and Technology, Clear Water Bay, Kowloon, Hong Kong SAR, 00000, China; Hong Kong Branch of Southern Marine Science and Engineering Guangdong Laboratory (Guangzhou), Hong Kong University of Science and Technology, Clear Water Bay, Kowloon, Hong Kong SAR, 00000, China; State Key Laboratory of Molecular Neuroscience, The Hong Kong University of Science and Technology, Clear Water Bay, Kowloon, Hong Kong SAR, 00000, China

## Abstract

The hexanucleotide repeat expansion, GGGGCC (G4C2), within the first intron of the *C9orf72* gene is known to be the most common genetic cause of both amyotrophic lateral sclerosis (ALS) and frontotemporal dementia (FTD). The G4C2 repeat expansions, either DNA or RNA, are able to form G-quadruplexes which induce toxicity leading to ALS/FTD. Herein, we report a novel crystal structure of d(G4C2)_2_ that self-associates to form an eight-layer parallel tetrameric G-quadruplex. Two d(G4C2)_2_ associate together as a parallel dimeric G-quadruplex which folds into a tetramer via 5′-to-5′ arrangements. Each dimer consists of four G-tetrads connected by two CC propeller loops. Especially, the 3′-end cytosines protrude out and form C·C+•C·C+/ C·C•C·C+ quadruple base pair or C•C·C+ triple base pair stacking on the dimeric block. Our work sheds light on the G-quadruplexes adopted by d(G4C2) and yields the invaluable structural details for the development of small molecules to tackle neurodegenerative diseases, ALS and FTD.

## INTRODUCTION

Amyotrophic lateral sclerosis (ALS) and frontotemporal dementia (FTD) are fatal degenerative neurological diseases, the former characterized by selective loss of motor neurons in the brain and spinal cord and the latter characterized by selective atrophy of frontal and temporal lobes, which have similar genetic and pathological background demonstrated by growing evidence from clinical, pathological and genetic findings (1–4). Extensive studies have identified that the aberrantly expanded hexanucleotide repeat GGGGCC (HRE G4C2) located in the first non-coding region of *C9orf72* gene is the most common genetic cause of ALS and FTD (5–7), and tremendous progress toward understanding disease mechanisms and developing therapies for ALS/FTD has been made (8–10). Especially, it is found that the *C9orf72* HRE G4C2 DNA/RNA sequence can fold into various complex secondary structures such as G-quadruplexes (11–15) and may be involved in the pathogenesis of ALS/FTD through a distinct mechanism associated with their structure polymorphism (16,17). Consequently, the high-resolution structural information of the diverse *C9orf72* HRE G4C2 G-quadruplexes is vital for understanding the mechanism of ALS/FTD and developing more effective therapeutic agents (9,18).

G-quadruplexes are stable four-stranded DNA helical structures that can form within G-rich DNA and RNA sequences (19). It can assemble from the same (intramolecular) or different (intermolecular) nucleic acid chains (20). The basic structural unit is the G-tetrad, in which four guanines form a cyclic Hoogsteen hydrogen-bonded square planar structure (21). Two or more G-tetrads stack to form a G-quadruplex and are stabilized by monovalent cations (22). The backbone strands of the G-quadruplex can adopt different orientations, such as parallel, antiparallel and hybrids (20,23).

Recently, great efforts have been devoted to investigate *C9orf72* HRE DNA G-quadruplexes (13,24–27). The *C9orf72* HRE DNA with different lengths including d(G4C2)G4, d(G4C2)_2_, d(G4C2)_3_, d(G4C2)_4_ and d(G4C2)_5_ was shown to adopt different G-quadruplex topologies by circular dichroism (CD) and NMR spectroscopy (Supplementary Table S1). Especially, previous findings suggested that the parallel topology is adopted by most *C9orf72* (G4C2)_n_ DNAs (13,26,27). However, due to the heterogeneous conformations of G4C2 oligonucleotides repeat, no parallel structure of *C9orf72* HRE DNA has been determined to date.

In previous studies, we have obtained a crystal of *C9orf72* HRE DNA sample, d(G4C2)_2_, which adopts the parallel topology and has sufficient quality for structural determination in K^+^ solution by X-ray crystallography (28). Unfortunately, molecular replacement method cannot provide a satisfactory solution with the use of a variety of known G-quadruplex structures in PDB (www.rcsb.org) including mono-, di- and tetrameric G-quadruplexes as well as G-quadruplexes with two, three and four G-quartets without connecting loops. However, it has been shown that the coordination of monovalent or divalent cations in the G-quadruplex has a great impact on the stability and polymorphism of G-quadruplex structures (29). Some divalent cations such as Ba^2+^, Pb^2+^ and Ca^2+^ in the central ion channel promote G-quadruplex folding in a number of specific cases (30–34). Particularly, the radius of Ba^2+^ (1.35 Å) is almost same as the radius of K^+^ and SAD signal of Ba^2+^ can be utilized to solve the phase problem in X-ray crystallography. Therefore, we tried to determine the structure of G4C2 by annealing the d(G4C2)_2_ DNA sample in Ba^2+^ solution at first. Strikingly, a parallel G-quadruplex was formed in Ba^2+^ solution and two crystal structures in different space groups were determined successfully by using the SAD signal of Ba^2+^. Subsequently, the structure was employed as the initial model for solving the crystal structure of d(G4C2)_2_ in K^+^ solution by molecular replacement.

Here, we present the first crystal structures of an eight-layer parallel tetrameric G-quadruplex formed by two repeats of *C9orf72* HRE DNA, d(G4C2)_2_, in Ba^2+^ and K^+^ solution, named as d(G4C2)_2_-Ba and d(G4C2)_2_-K respectively. Two d(G4C2)_2_ oligonucleotides form a parallel propeller-type dimeric G-quadruplex which stacks in a 5′-to-5′ arrangement making up a tetramer. Intriguingly, two kinds of the 5′-to-5′ orientation, Form-1/1 and Form-1/7, were observed in the crystal G-quadruplex structures of d(G4C2)_2_-Ba and d(G4C2)_2_-K respectively. One of the dimeric G-quadruplex in Form-1/1 (the G1 base in one dimeric block stacks with the G1 base in the opposite dimeric block) rotates 90° compared with the corresponding dimeric structure in Form-1/7 (the G1 base in one dimeric block stacks with the G7 base in the opposite dimeric block). Each dimeric G-quadruplex is composed of four G-tetrads connected by two CC double-chain-reversal loops. The two cytosine bases located at the 3′ end of each stand protrude out to form C·C+•C·C+/ C·C•C·C quadruple base pair or C•C·C+ triple base pair that stacks on the neighbouring dimeric blocks. Atomic details of the G4 structure discovered in our work shed light on the structural diversity of G-quadruplexes adopted by d(G4C2) repeats and allow us to design small molecules to modulate aberrant transcription of *C9orf72* gene related with ALS and FTD in particular by *in silico* drug screening studies.

## MATERIALS AND METHODS

### Sample preparation

The single DNA strands were purchased from Integrated DNA Technologies (IDT) and Takara. For the sample containing Ba^2+^, the oligonucleotide was dissolved in buffer containing 10 mM BaCl_2_ and 20 mM Tris (pH 7.0) at 0.1 mM (single strands) and annealed by heating to 95°C for 15 min, followed by slow cooling to room temperature overnight. Then, the DNAs were concentrated for crystallization.

For the sample containing K^+^, the oligonucleotide was annealed in buffer containing 70 mM KCl and 20 mM potassium phosphate (pH 7.0) at 0.1 mM (single strands). Then, the DNAs were purified by FPLC using the Mono-Q column (GE healthcare) following the protocol in the previous report (28). The fifth fraction of the elution peaks from the Mono-Q column was collected and buffer exchanged into 20 mM Tris, 100 mM KCl buffer at pH 7.0 by desalting column. The DNAs were further concentrated for crystallization. The fifth fraction of the elution peaks from the Mono-Q column was exchanged into buffer containing 20 mM potassium phosphate, 100 mM KCl buffer at pH 7.0 by desalting column for CD, NMR and PAGE experiments.

### NMR spectroscopy

Nuclear magnetic resonance (NMR) experiments were conducted on 500 and 800 MHz Varian spectrometers at 25°C. The concentration of DNA samples was typically ∼0.1 mM.

### Circular dichroism spectroscopy

Circular dichroism (CD) spectra were recorded on an Applied Photophysics Chirascan CD spectrometer at 25°C using 1 mm path length quartz cuvette with sample volume 400 μl. The DNA oligonucleotides were prepared at concentration of 15 μM (single strands).

### Polyacrylamide gel electrophoresis (PAGE)

Non-denaturing PAGE was carried out in 20% polyacrylamide gel (acrylamide:bis-acrylamide 29:1), supplemented with 20 mM KCl in the gel and running buffer (0.5× TBE). The samples were prepared at a single strand concentration of 100 μM. Gels were stained by red-safe dye.

### Crystallization

The DNA samples at concentration of ∼1.8–2 mM in Ba^2+^ and K^+^ solution were initially screened using Nucleic Acid Mini Screen Kit and Natrix Kit (Hampton Research) with the hanging-drop vapor-diffusion technique at 16°C. After optimization, for d(G4C2)_2_-Ba (C222_1_), 1.07 mM DNA was crystallized in the final crystallization condition of 40 mM sodium cacodylate trihydrate pH 7.0, 10% MPD, 12 mM spermine tetrahydrochloride, 80 mM NaCl and 20 mM BaCl_2_ with the reservoir buffer of 17.5% MPD. For d(G4C2)_2_-Ba (F222), 1.07 mM DNA was crystallized in the final crystallization condition of 40 mM sodium cacodylate trihydrate pH 7.0, 10% MPD, 12 mM spermine tetrahydrochloride, 80 mM KCl and 20 mM MgCl_2_ with the reservoir buffer of 17.5% MPD. For d(G4C2)_2_-K (F222), 1 mM DNA was crystallized in the final crystallization condition of 50 mM sodium cacodylate trihydrate pH 6.0, 45% MPD, 12 mM spermine tetrahydrochloride, 80 mM KCl and 20 mM MgCl_2_ with the same reservoir buffer.

### Data collection and structure determination

For data collection, the crystals of d(G4C2)_2_-Ba and d(G4C2)_2_-K were directly flash-cooled in liquid nitrogen. The diffraction data sets were collected on beamlines BL17U and BL19U at Shanghai Synchrotron Radiation Facility (SSRF) at the wavelengths as indicated in Supplementary Table S2. Intensity data were integrated and scaled by HKL2000 and HKL3000 packages (35).

The structure of d(G4C2)_2_-Ba in C222_1_ space group (d(G4C2)_2_-Ba (C222_1_)) was firstly determined by single wavelength anomalous dispersion (SAD) method. The positions of Ba^2+^ atoms were found and refined, then the phases were calculated using SHELX (36) (Supplementary Figure S1). Manual model building and refinement were performed iteratively with COOT (38), Refmac5 (39) and phenix.refine (40). The initial phases of the structure of d(G4C2)_2_-Ba in F222 space group (d(G4C2)_2_-Ba (F222)) were got by molecular replacement method using a d(G4C2)_2_ chain from the d(G4C2)_2_-Ba (C222_1_). Then, we combined initial phases by molecular replacement and SAD to get the Ba positions and SAD map for the following modeling using Phaser-EP (37) (Supplementary Figure S2). After manual model building and refinement, the structure of d(G4C2)_2_-Ba (F222) was employed as template to solve the structure of d(G4C2)_2_-K by molecular replacement method using Phaser (37). Further manual model building and refinement were performed iteratively with COOT (38), Refmac5 (39) and phenix.refine (40). The final refinement statistics were summarized in Supplementary Table S2. All figures of G-quadruplex structure were prepared using PyMOL (http://www.pymol.org).

## RESULTS

### Parallel G-quadruplex formed by d(G4C2)_2_ in Ba^2+^ solution

**Figure 1.**
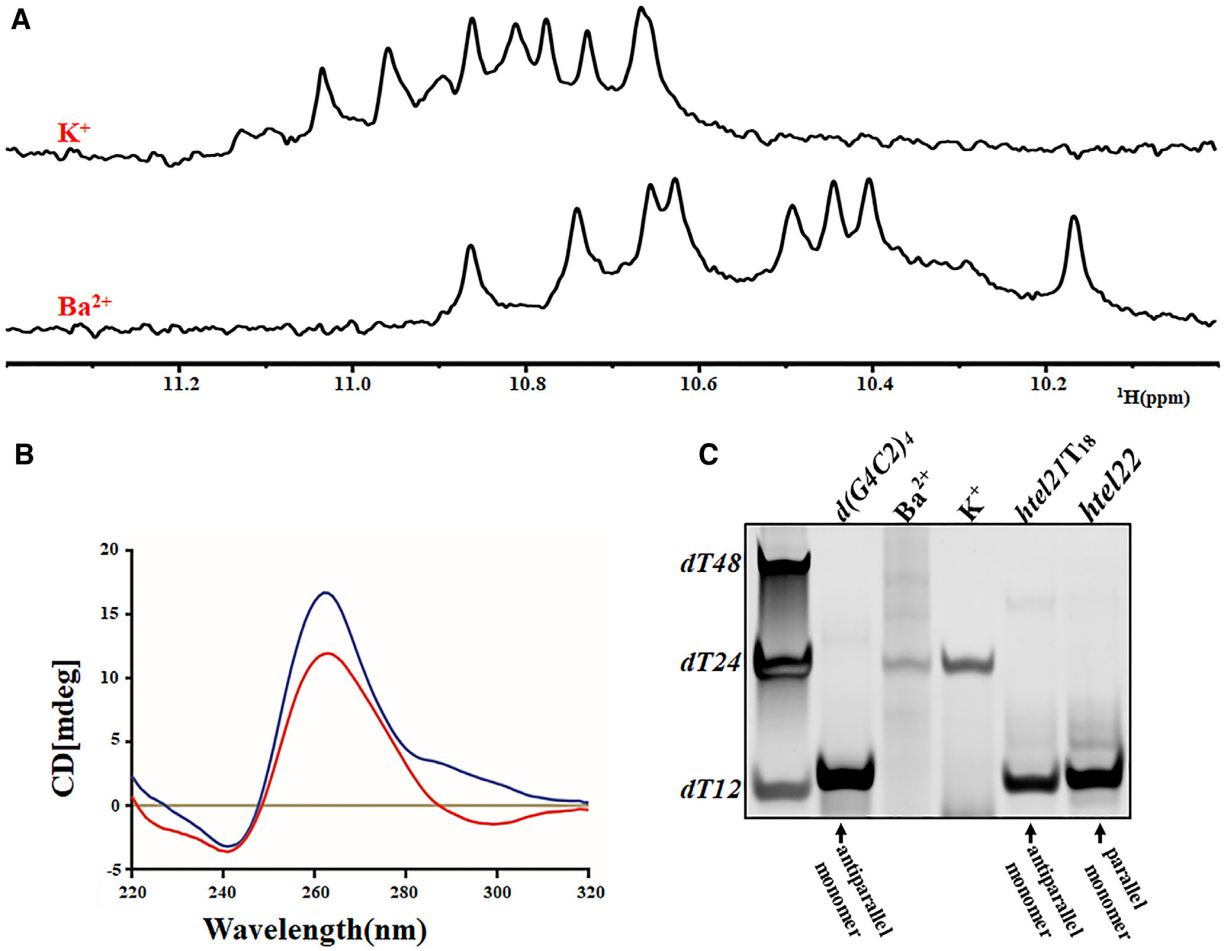
Characterization of d(G4C2)_2_ in Ba^2+^ and K^+^ solutions. (**A**) The imino region of 1D ^1^H-NMR spectra of d(G4C2)_2_ in 20 mM potassium phosphate solution containing 70 mM KCl and 10mM BaCl_2_ with pH 7.0 recorded at 25°C on 800 MHz, respectively. (**B**) CD spectra in 20 mM potassium phosphate solution containing 10mM BaCl_2_ (red) and 70 mM KCl (blue) with pH7.0 recorded at 25°C, respectively. (**C**) Electrophoretic mobility in non-denaturing 20% PAGE at 100 µM concentration of d(G4C2)_2_ with other references: d(G4C2)_4_ (d[(GGGGCC)_4_]), a monomeric four-layer antiparallel G-quadruplex (26); *htel21*T_18_ (d[(GGGTTA)_2_GGGTTTGGG]), a monomeric three-layer antiparallel G-quadruplex (43), and *htel22* (d[A(GGGTTA)_3_GGG]) a monomeric three-layer parallel G-quadruplex (44).

d(G4C2)_2_ can form a mixture of G-quadruplex conformations in the K^+^ solution including parallel and parallel/antiparallel folds which can be separated by anion exchange chromatography (28). However, the heterogeneity of d(G4C2)_2_ makes structural elucidation difficult. Interestingly, it was reported that divalent cations can induce a transition from an antiparallel to a parallel G-quadruplex conformation (30,41,42). Based on the fact that only the radius of Ba^2+^ (1.35 Å) is same as that of K^+^ (1.33 Å) when compared with other known divalent cations such as Pb^2+^, Sr^2+^, Ca^2+^ used in the study of G-quadruplex. We chose to study d(G4C2)_2_ in the Ba^2+^ solution. Remarkably, the 1D ^1^H NMR spectra of the d(G4C2)_2_ in K^+^ and Ba^2+^ solution showed ∼8 well-resolved imino proton resonances at 10–11.2 ppm respectively (Figure 1A), clearly indicating the formation of a predominant G-quadruplex structure in each solution. Notably, circular dichroism (CD) spectrum of d(G4C2)_2_ in Ba^2+^ solution exhibits the similar profile as the one in K^+^ solution with the general features of a typical parallel G-quadruplex, characterized by a dominant positive peak at ∼260 nm and a negative peak at ∼240 nm (Figure 1B).

To probe the molecular sizes of d(G4C2)_2_ in K^+^ and Ba^2+^ solution, we performed gel electrophoresis experiments using references including DNA oligonucleotides dT12, dT24 and dT48, d(G4C2)_4_ (a monomeric 24 bp four-layer antiparallel G-quadruplex) (26), *htel21*T_18_ (a monomeric 21 bp three-layer antiparallel G-quadruplex) (43) and *htel22* (a monomeric 22 bp three-layer parallel G-quadruplex) (44).

As shown in Figure 1C, all the monomeric G-quadruplexes d(G4C2)_4_, *htel21*T_18_ and *htel22* migrated similarly as dT12. However, the migration of d(G4C2)_2_ in K^+^ and Ba^2+^ solution is slower than dT12 and comparable to dT24, indicating formation of multimeric structures and potentially a tetrameric G-quadruplex in solution. This conclusion was further supported by the crystal structures (see below).

### Crystal structure of tetrameric parallel G-quadruplexes formed by d(G4C2)_2_ in Ba^2+^ solution

We successfully crystallized d(G4C2)_2_-Ba in two different space group, C222_1_ and F222. The crystal structure of d(G4C2)_2_-Ba in C222_1_ space group was solved by Ba-SAD method to 1.60 Å resolution (Supplementary Table S2 and Supplementary Figure S1). The crystal structure of d(G4C2)_2_-Ba in F222 space group was solved by a combination of molecular replacement and SAD method (Supplementary Table S2 and Supplementary Figure S2). Since the structures of d(G4C2)_2_-Ba in F222 space group and d(G4C2)_2_-K share similar features, we will discuss them later.

**Figure 2.**
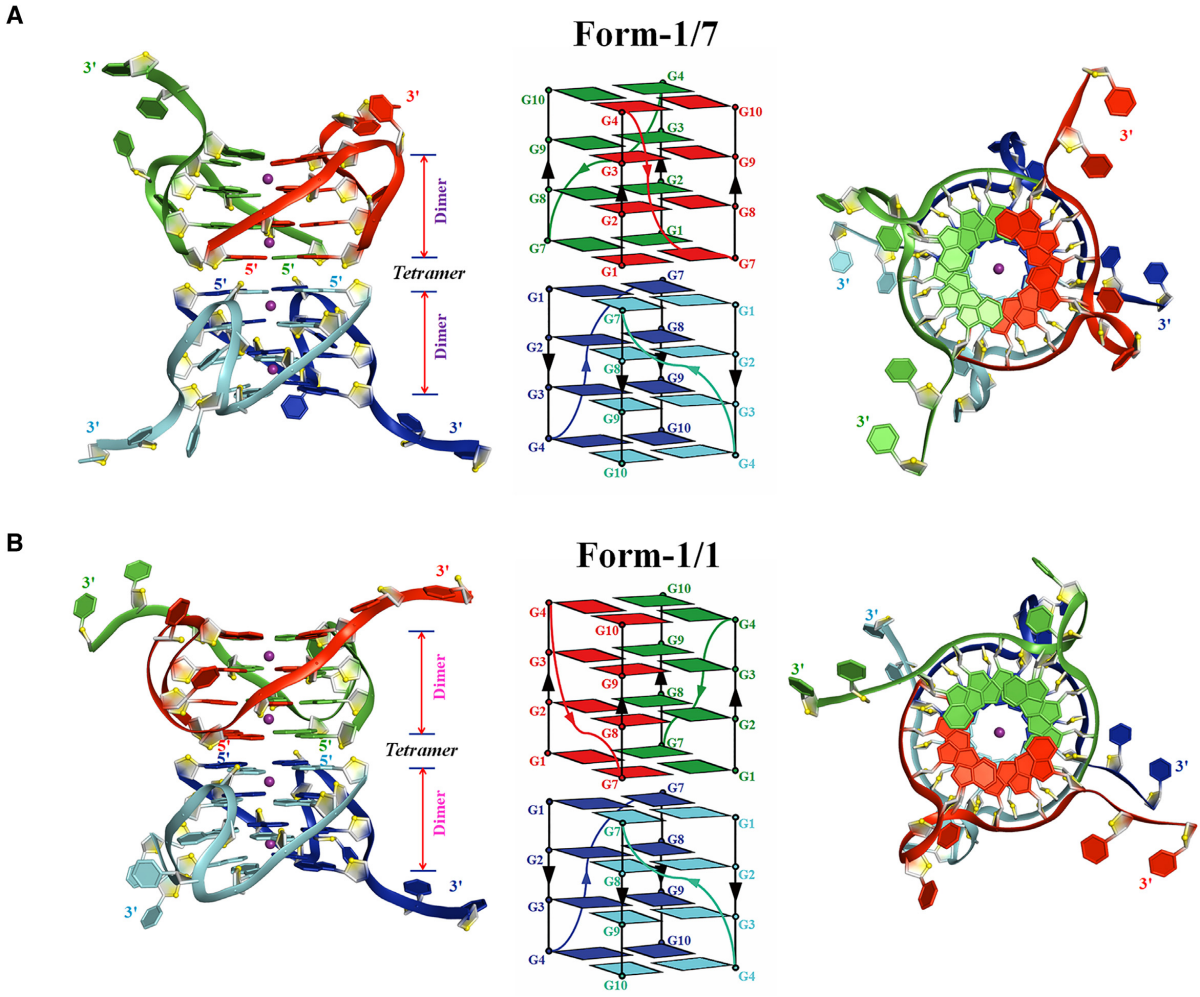
Crystal structure of d(G4C2)_2_-Ba in the space group of C222_1_. (**A**) Form-1/7 formed by chains A/B and their crystallographically symmetric chains A_sym_/B_sym_. (**B**) Form-1/1 formed by chains E/F and their crystallographically symmetric chains E_sym_/F_sym_. Each dimeric block is stacked to form a tetrameric G-quadruplex via different 5′-arrangments and stabilized by Ba^2+^ (purple sphere). Left: Cartoon representation of tetrameric G-quadruplex formed by d(G4C2)_2_. Middle: Schematic representation of topology adopted by d(G4C2)_2_. Right: Top view of Left. Each molecule, d(G4C2)_2_, is shown as red, green, blue and cyan in the tetrameric G-quadruplex. O4′ oxygens are in yellow.

For the structure of d(G4C2)_2_-Ba (C222_1_), the asymmetric unit contains six chains, A–F (Supplementary Figure S3A). Intriguingly, two d(G4C2)_2_ oligonucleotides form a parallel-stranded dimeric G-quadruplex unit (i.e. chains A/B, chains C/D, chains E/F) which is composed of four G-tetrads connected by two CC double-chain-reversal loops. The dimeric G-quadruplex unit co-axially stacks on the other crystallographically symmetric dimeric G-quadruplex unit in a 5′-to-5′ arrangement resulting in tetramer through π–π interactions with two distinguish stacking (Figure 2). Specifically, chains A/B and their crystallographically symmetric molecules (chains A_sym_/B_sym_) form a parallel-stranded tetrameric eight-layer G-quadruplex termed as Form-1/7 with the G1 base in one dimeric block stacks (i.e. chains A/B) with the G7 base in the opposite dimeric block (i.e. chains A_sym_/B_sym_) (Supplementary Figures S3B, S4A and Figure 2A). While another two identical parallel-stranded tetrameric G-quadruplexes are formed by the chains C/D with their crystallographically symmetric molecules (chains C_sym_/D_sym_) or chains E/F with their crystallographically symmetric molecules (chains E_sym_/F_sym_). Both of them are named as Form-1/1 as the G1 base of one dimeric block (i.e. chains C/D or chains E/F) stacks on the G1 base of another dimeric block (i.e. chains C_sym_/D_sym_ or chains E_sym_/F_sym_) (Supplementary Figures S3B, S4B and Figure 2B). Two tetrameric G-quadruplexes with Form-1/1 share the same folding with RMSD of core guanine residues of 1.90 Å.

In each dimeric G-quadruplex unit, the two cytosine bases located at 3′ end of each stand protrude and stack on the neighbouring dimeric block (see below). The hydrogen-bond directionalities of the four G-tetrads in each dimeric block are clockwise (G1→G7→G1′→G7′, G2→G8→G2′→G8′, G3→G9→G3′→G9′ and G4→G10→G4′→ G10′ with the prime (′) signifying the bases belong to separate oligonucleotide strands in the same dimeric block) with all Hoogsteen N1-O6 and N2-N7 hydrogen bonds intact. The glycosidic conformations of all bases in each G-quadruplex are *anti*.

### The Ba^2+^ in the structure of d(G4C2)_2_-Ba

The electron density was well defined for the four equal-spaced Ba^2+^ ions lying along the axis within the central core of the eight-layer tetrameric G-quadruplex in Form-1/7 and Form-1/1 (Figure 2 and Supplementary Figure S5). It is unique that the tetrameric G-quadruplex is associated with divalent Ba^2+^ ions between every other guanine tetrad plane to balance charges. Each of them is coordinated to eight neighbouring guanine O6 atoms at a distance of ∼2.7 Å, featuring an anti-prismatic coordination environment, which mimics those of monovalent potassium ions in DNA G-quadruplexes. The structural architecture of central Ba^2+^ ions is similar as the one in G-quadruplex structure formed by, d(CCAC^NVK^GCGTGG), in the presence of Ba^2+^ (45). However, the later one contains a water-mediated C-tetrad which may stabilize the adjacent central divalent cation.

Interestingly, an additional Ba^2+^ ion is positioned between two adjacent dimeric G-quadruplexes (formed by chains A/B and chains C/D, respectively) in the asymmetric unit (Supplementary Figure S3A). This ion is observed in direct contact (<3.0 Å) with six additional water molecules. The network forming by Ba^2+^ ion and water molecules bridges between the phosphate group of C6, the N2 atom of G4 and the phosphate group of G9, the N2 and N3 atoms of G4 belonging to two adjacent G-quadruplexes respectively (Supplementary Figure S6).

### The cytosine conformations in the structure of d(G4C2)_2_-Ba

**Figure 3.**
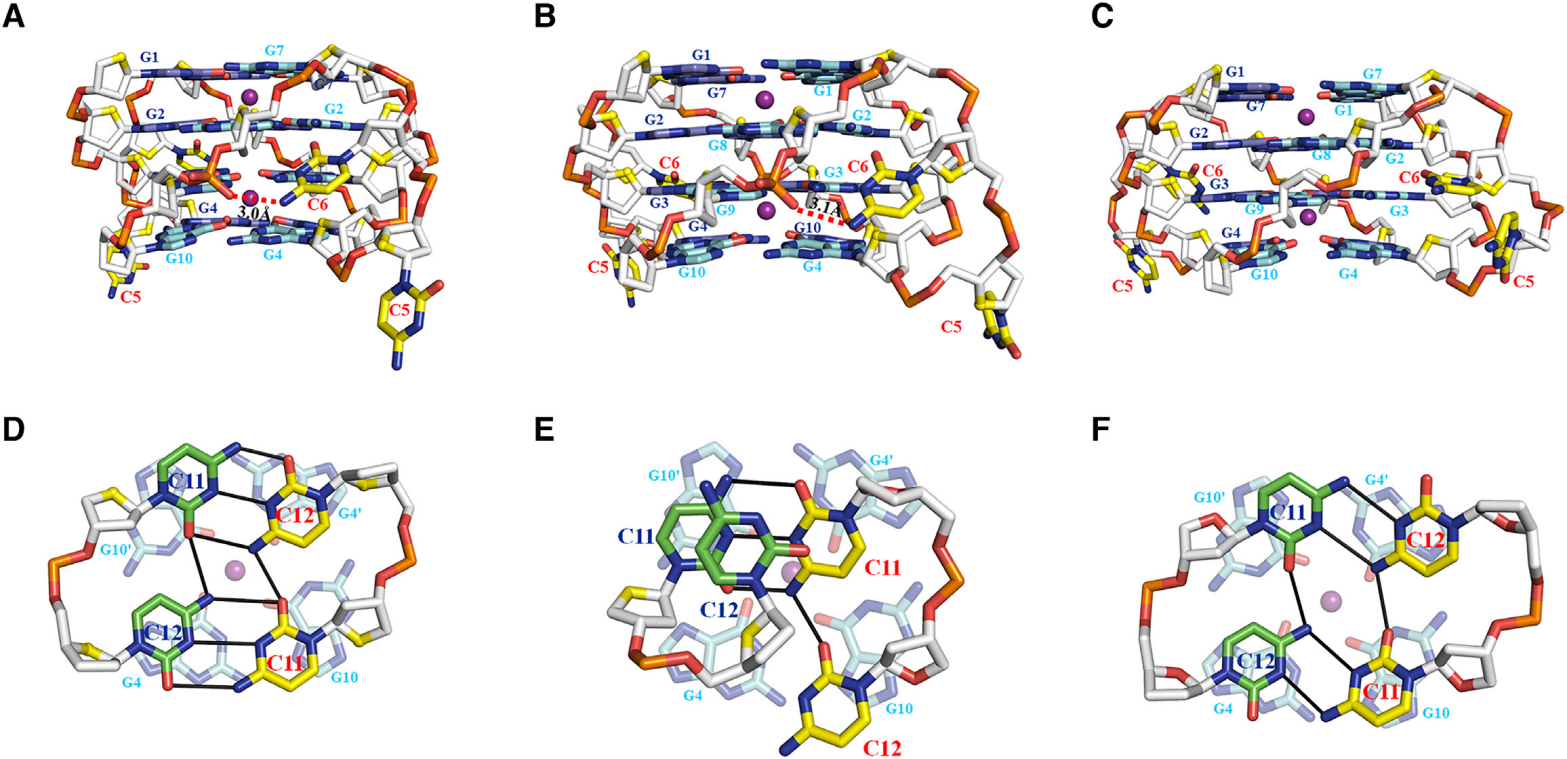
Detailed conformations of cytosines in the tetrameric G-quadruplex formed by d(G4C2)_2_-Ba (C222_1_). The conformation of propeller loop, C5 and C6, in the dimeric G-quadruplex of (**A**) Form-1/7 formed by chains A/B, (**B**) Form-1/1 formed by chains C/D and (**C**) Form-1/1 formed by chains E/F. The conformation of the C11 and C12 bases of (**D**) chain A (green) and chain F (yellow), (**E**) chain B (yellow) and chain D (green) and (**F**) chain C (yellow) and chain E (green) located at the 3′- end observed in the unit cell. The hydrogen bonds are represented by read dash and solid black lines.

In each dimeric G-quadruplex unit of Form-1/7 and Form-1/1, the four G-tetrads are connected by two propeller loops composed of C5 and C6 bases (Figure 2). However, the C5-C6 loop regions show some degree of disorder, exemplified by weak electron density (Supplementary Figure S7). The position of the observed C5 bases inserts the medium groove of the four-layer G-tetrad core and all observed C6 bases sit outside of the four-layer G-tetrad core (Figure 3A–C and Supplementary Figure S7). Specifically, the N4 atom of C6 and the phosphate oxygen group of G9 form a hydrogen bond (Figure 3A and B). However, this hydrogen bond is missing in the Form-1/1 formed by chains E/F (Figure 3C), indicating the flexibility of C5-C6 loop regions.

All the C11 and C12 bases located at the 3′ end are well defined (Supplementary Figure S7). The C11 and C12 bases protrude out and position outside the G-tetrad core in tetrameric Form-1/7 and Form-1/1 (Figure 2 and Supplementary Figure S6).

Interestingly, the C11 and C12 bases from two isolated chains, chains A and F, form a C·C+•C·C+ quadruple base pair which stacks on a G-tetrad layer of Form-1/7, G4·G10·G4′·G10′, belonging to the neighbouring tetrameric G-quadruplex (Figure 3D). Whereas the C11 and C12 base of chain B and the C11 base of chain D form a C-triad containing a C·C+ base pair connected with the C11 base of chain B through a C12N4-C11O2 hydrogen bond which stacks on a G-tetrad layer of Form-1/1, G4·G10·G4′·G10′, belonging to the neighbouring tetrameric G-quadruplex (Figure 3E). The C12 base of chain D parallels with the C-triad. Strikingly, the C11 and C12 bases from two isolated chains, chains C and E, form a C·C•C·C quadruple base pair which stacks on a G-tetrad layer of Form-1/7, G4·G10·G4′·G10′, belonging to the neighbouring tetrameric G-quadruplex (Figure 3F).

### Crystal structure of tetrameric parallel G-quadruplexes formed by d(G4C2)_2_ in K^+^ solution

The structure of d(G4C2)_2_ in K^+^ solution, d(G4C2)_2_-K, was solved via molecular replacement using the crystal structure of d(G4C2)_2_-Ba (F222) as searching model. The space group of d(G4C2)_2_-K is also F222 with similar cell dimensions (Supplementary Table S2), indicating they are isomorphous. Both d(G4C2)_2_-Ba (F222) and d(G4C2)_2_-K (F222) share similar structural features (with RMSD of core guanine residues of 0.243 Å) except the ions, thus we describe them here together.

**Figure 4.**
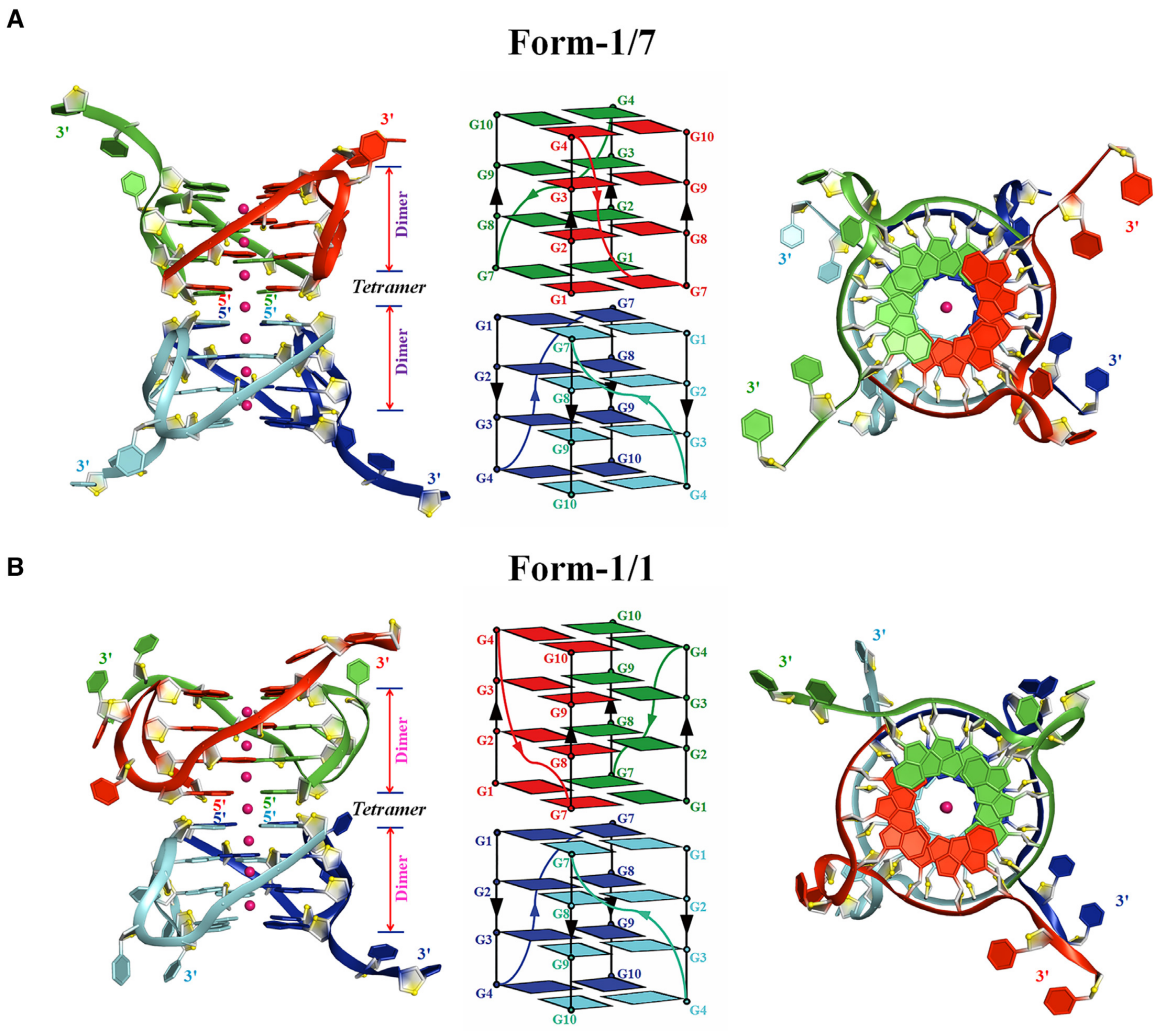
Crystal structure of d(G4C2)_2_-K in the space group of F222. (**A**) Form-1/7 and (**B**) Form-1/1 in which each dimeric block is stacked to form a tetrameric G-quadruplex via different 5′-arrangments and stabilized by K^+^ (magenta sphere). Left: Cartoon representation of tetrameric G-quadruplex formed by d(G4C2)_2_. Middle: Schematic representation of topology adopted by d(G4C2)_2_. Right: Top view of Left. Each molecule, d(G4C2)_2_, is shown as red, green, blue and cyan in the tetrameric G-quadruplex. O4′ oxygens are in yellow.

There are three chains, A-C in an asymmetric unit (Supplementary Figure S7A). Similar with the d(G4C2)_2_-Ba (C222_1_), two kinds of parallel-stranded tetrameric eight-layer G-quadruplexes were observed. The Form-1/7 stacking form was composed by chain A and three other crystallographically symmetric chains A_sym1_/A_sym2_/A_sym3_ for both d(G4C2)_2_-Ba (F222) and d(G4C2)_2_-K (F222). The Form-1/1 stacking form was composed by chains B/C and their crystallographically symmetric chain B_sym_/C_sym_ (Supplementary Figures S8, S9, S10 and Figure 4). Notably, there are seven equal-spaced K^+^ ions lying along the axis within the central core of the tetrameric G-quadruplex including a well-defined central channel potassium ion located in the interface between the two dimeric blocks (Figure 4 and Supplementary Figure S11), while only four equal-spaced Ba^2+^ ions lying along the axis in the structure of d(G4C2)_2_-Ba (F222), which is exactly the same as the Ba^2+^ ions in the structure of d(G4C2)_2_-Ba (C222_1_) (Supplementary Figure S10).

### The cytosine conformations in the structure of d(G4C2)_2_-K

**Figure 5.**
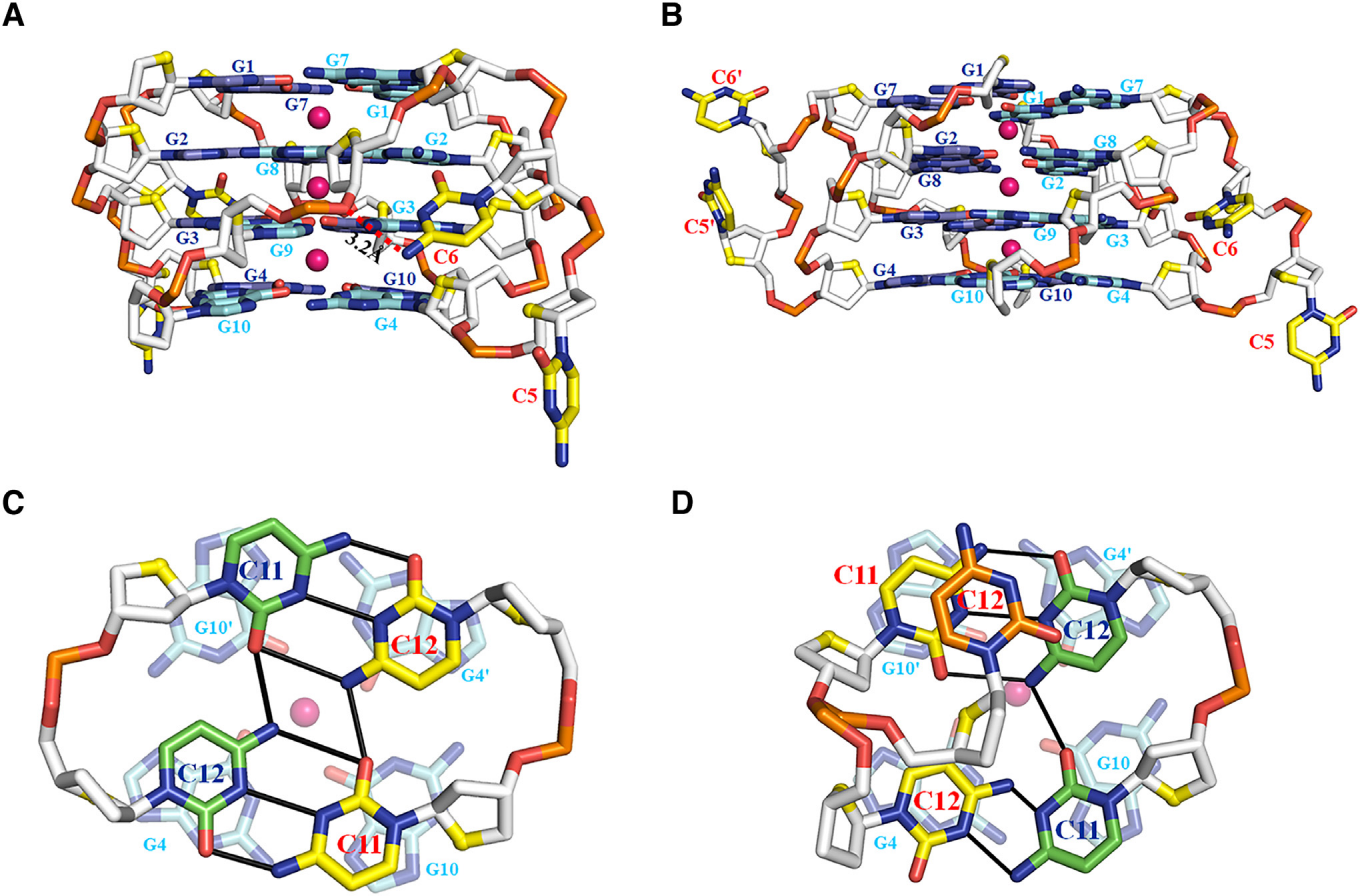
Detailed conformations of cytosines in the tetrameric G-quadruplex formed by d(G4C2)_2_ in K^+^ (magenta sphere). The conformation of propeller loop, C5 and C6, in each tetrameric G-quadruplex of (**A**) Form-1/7 and (**B**) Form-1/1. (**C**) The conformation of the C11 and C12 bases of chain C forming Form-1/1 located at the 3′- end in the unit cell. (**D**) The conformation of the C11 and C12 bases of chain A (green) forming Form-1/1 and B (yellow) forming Form-1/7. Another conformation observed for the C12 base of chain B is colored by orange. The hydrogen bonds are represented by red dash and solid black lines.

In the Form-1/7, the C6 base is well defined and inserts the groove of the four-layer G-tetrad core (Figure 5A and Supplementary Figure S12A). Interestingly, a hydrogen bond formed by the N4 atom of C6 and the O3′ atom of G8 is observed (Figures 5A). However, the electron density clearly indicates that the C5 sits outside of the G-tetrad core of Form-1/7 (Figure 5A and Supplementary Figure S12A). The C5-C6 loop regions in the Form-1/1 show some degree of disorder in the electron density and protrude out except the well-defined C6 base of chain B sitting in the medium groove of Form-1/1 (Figure 5B and Supplementary Figure S12B).

The C11 and C12 bases in Form-1/7 and Form-1/1 are well defined and protrude out except the C12 base of chain B in Form-1/1 showing two alternative conformations (Supplementary Figure S12B). The C11 and C12 bases from chains C and C_sym_ of Form-1/1 form a C·C+•C·C+ quadruple base pair which stacks on a G-tetrad layer of Form-1/7, G4·G10·G4′·G10′, belonging to the neighbouring tetrameric G-quadruplex (Figure 5C). Interestingly, the well-defined C11 and C12 bases of chain A and the C11 base of chain B form a C-triad containing a C·C+ base pair connected with a cytosine through a C12N4-C11O2 hydrogen bond (Figure 5D). Intriguingly, the C12 of chain B associates with the C-triad through (i) forming a C·C base pair with the C11 of Chain A which results in a C·C•C·C+ quadruple base pair; (ii) π–π interactions stacking on the C-triad (Figure 5D).

## DISCUSSION

Here, we showed that two d(G4C2)_2_ oligonucleotides form a parallel propeller-type dimeric G-quadruplex which co-axially stacks in a 5′-to-5′ arrangement making up an eight-layer parallel tetramer in Ba^2+^ and K^+^ solution. Although the structure of d(G4C2)_2_ in Ba^2+^ solution is almost identical to the one in K^+^ solution, the different chemical shift dispersion of imino protons is possibly caused by the unique arrangement of Ba^2+^ and K^+^ within the central channel in each structure. Interestingly, a non-central channel Ba^2+^ is observed without close contact to DNA strand and water. The crystal structures of d(G4C2)_2_-Ba in different space groups (i.e. C222_1_ and F222) have similar architecture, with the fact that the RMSD values of core guanine residues of Form-1/7 and Form-1/1 are 0.49 and 0.34 Å, respectively. The conformation of CC double-chain-reversal loops in d(G4C2)_2_-Ba^2+^ (F222) is also similar as d(G4C2)_2_-Ba^2+^ (C222_1_) and C·C+•C·C+ quadruple base pair and C•C·C+ triple base pair formed by the the 3′ end are also observed (Supplementary Figure S13) that stacks on the neighbouring dimeric blocks. Furthermore, not surprisingly, additional intermolecular π−π packing interactions for cytosine bases were observed in Ba^2+^ and K^+^ solution (Supplementary Figure S14).

Until now, only two G-quadruplex structures formed by d(G4C2)_4_ repeats have been reported. The d(G4C2)_4_ repeats forms two monomeric four-layer antiparallel G-quadruplexes which differ in the donor–acceptor directionalities for each individual hydrogen-bonded pair in the G-quartets (25,46). The antiparallel G-quadruplex adopted by d(G4C2)_4_ contains three lateral CC loops, where the cytosines form a pseudo C-quartet containing two independent C·C/C·C+ base pair stacking on the nearby G-tetrad. However, quadruple base pair, C·C+•C·C+/C·C•C·C/C·C•C·C+, and triple base pair, C•C·C+, stacking on the neighbouring G-tetrad are observed in our structures. The quadruple base pair are not observed in antiparallel form which is possibly due to the intra cytosines forming C·C/C·C+ base pair. Whereas all cytosines in our case are inter which come from neighboring independent G-quadruplexes. It was also reported that a C-tetrad, stabilized by hydrogen bonds formed by atoms O2 and N4 in cytosine, was generally observed in parallel G-quadruplexes adopted by the DNA sequences such as d(CCAC^NVK^GCGTGG) (45), d(TG3CG2T) from SV40 virus (47), d(TG2CG2C) from Fragile X syndrome (48) and d(AGAGAGATGGGTGCGTT) (49). However, C-tetrad is not observed in our case implying the quadruple base pair in which C·C+/C·C base pairs are connected by hydrogen bonds are more stable than C-tetrad and can further stabilize G-quadruplex. The formation of the C·C+ base pair, the hemiprotonated cytosine·cytosine, generally are observed in I-motif structures and occurs at the acidic pH, recent studies demonstrated that formation of the C·C+ base pair could occur at neutral pH by *in vitro* and *in vivo* experiments (50,51). Notably, since the d(G4C2)_2_ sequence was crystalized at pH 7.0, our result provides a structural evidence for the existence of the C·C+ base pair at neutral pH. Although the *C9orf72* HRE DNA with different lengths can adopt different G-quadruplex topologies including antiparallel, parallel and mixed parallel/antiparallel types (Supplementary Table S1), potentially these different topologies are in a dynamic equilibrium resulting in different higher order quadruplex structures (42,52,53).

The formation of G-quadruplex by *C9orf72* HRE DNA, d(G4C2)_n_, has been reported to impair RNA polymerase processivity leading to an increase in abortive RNA transcripts which result in the pathogenesis of *C9orf72* linked ALS/FTD(12). It has also been suggested that *C9orf72* d(G4C2)_n_ in ALS/FTD can form a large ‘G-quadruplex island’ in which each individual G-quadruplex adopting an antiparallel conformation (54). However, our crystal structure indicates that DNA G-quadruplexes in ‘G-quadruplex island’ trend to adopt a parallel conformation through 5′-to-5′/3′-to-3′ stacking resulting in high-order G-quadruplex structures. Furthermore, in ALS/FTD, (G4C2)_n_ RNA transcripts which can form G-quadruplex accumulate in RNA foci in the nucleus, sequestering several RNA-binding proteins (54). However, no high-resolution structure of RNA (G4C2)_n_ is reported except molecular dynamics (MD) study based on human telomeric r(GGGTTA)_n_ structure which contains three G-tetrad layers (55). For the first time, we presented the crystal structure of the parallel tetrameric G-quadruplex adopted by the two G4C2 repeats DNA in which two CC propeller loops span four G-tetrad layers respectively. Our atomic resolution structures provide a more accurate structural model to study the G-quadruplex structure of r(G4C2)_n_ in ALS/FTD. Especially, the 5′-to-5′ stacking mode well explained the formation of RNA foci potentially caused by 5′- or 3′- end arranging mode resulting in the pathogenesis of ALS/FTD.

It has been demonstrated that *C9orf72* HRE G4C2 forms G-quadruplex structures *in vitro* and the structural polymorphism of *C9orf72* HRE G4C2, including G-quadruplex structures at both DNA and RNA level, is implicated in the development of ALS and FTD (56). Consequently, in light of pharmacological advantages, small molecules targeting G-quadruplexes formed by *C9orf72* HRE G4C2 DNA/RNA offer an attractive option as a therapeutic approach for ALS/FTD and several novel compounds have been reported in patient-derived neurons (9,57). Hence, it is important to explore the structural characteristics of *C9orf72* HRE G4C2 due to the potential roles of the G-quadruplex structures formed by *C9orf72* HRE G4C2 in the disease mechanism and therapy of ALS and FTD. Therefore, our unique high-resolution crystal structures either in Ba^2+^ or K^+^ solution reported here are invaluable for designing/improving highly selective and stable small molecules for potentially ameliorating the pathology of ALS and FTD in the future.

## DATA AVAILABILITY

Atomic coordinates and structure factors for the reported crystal structures of d(G4C2)_2_-Ba (C222_1_), d(G4C2)_2_-Ba (F222) and d(G4C2)_2_-K have been deposited with the Protein Data Bank under accession number 7ECF, 7ECG and 7ECH.

## Supplementary Material

gkab302_Supplemental_File
