# Supplementary material for: Crystal structure of parallel G-quadruplex formed by the two-repeat ALS- and FTD-related GGGGCC sequence
Source: Nucleic Acids Res. 2021 May 28;49(10):5881–90. doi: 10.1093/nar/gkab302 (PMC8191786; doi:10.1093/nar/gkab302)
Supplement: gkab302_Supplemental_File [file gkab302_supplemental_file.pdf]

Supporting Information

Crystal structure of parallel G-quadruplex formed by the two-repeat ALS and FTD related GGGGCC sequence

Yanyan Geng[1†], Changdong Liu[1†], Qixu Cai[1†], Zhipu Luo[2], Naining Xu[1], Chun Po Fung[1], Bing Yan[1], Ning Li[1], Peiyuan Qian[3], Bo Zhou[1, 4 *] and Guang Zhu[1, 3,5 *]

[1] Division of Life Science, The Hong Kong University of Science and Technology, Clear Water Bay, Kowloon, Hong Kong SAR, China
[2] Institute of Molecular Enzymology, School of Biology & Basic Medical Sciences, Soochow University, Suzhou, Jiangsu, China
[3] Hong Kong Branch of Guangdong Southern Marine Science and Engineering Laboratory (Guangzhou), Hong Kong University of Science and Technology, Clear Water Bay, Kowloon, Hong Kong SAR, China
[4] Institute for Advanced Study, The Hong Kong University of Science and Technology, Clear Water Bay, Kowloon, Hong Kong SAR, China
[5] State Key Laboratory of Molecular Neuroscience, The Hong Kong University of Science and Technology, Clear Water Bay, Kowloon, Hong Kong SAR, China

* To whom correspondence should be addressed. Tel: 852-2358-8705; Fax: (852)-2358-1552; Email: bzhou@alumni.ust.hk, gzhu@ust.hk

[†] The authors wish it to be known that, in their opinion, the first 3 authors should be regarded as joint First Authors.

Supplementary Tables and Figures

Table S1. The list of structural studies on the *C9orf72* d(G4C2)$_n$ DNAs.

Sequence	Topology	Method
d(G4C2)G4	*parallel* in K^+ solution, pH7.0[1]	CD spectroscopy
d(G4C2)$_2$[a*]	a *mixture* in K^+ solution, pH7.0[1-3]	CD, NMR spectroscopy and X-ray
d(G4C2)$_2$[b*]	*parallel* in Ba^{2+} solution, pH7.0	CD spectroscopy and X-ray
d(G4C2)$_3$	a *mixture* in K^+ solution, pH7.0[1]	CD spectroscopy
d(G4C2)$_4$[c*]	*antiparallel* in K^+ solution, pH7.0[1, 4-5]	CD and NMR spectroscopy
d(G4C2)$_5$	a *mixture* in K^+ solution, pH7.0[1]	CD spectroscopy

a*, b* In K^+ solution, we previously demonstrated that d(G4C2)$_2$ simultaneously can form two kinds of G-quadruplex folds, parallel and mixed parallel/antiparallel topologies, in which each fold can be separated by anion exchange chromatography[2]. The parallel G-quadruplex structures formed by d(G4C2)$_2$ in K^+ solution and Ba^{2+} are reported in this work.

c* The d(G4C2)$_4$ repeats, d[(G4C2)$_3$GG^{Br}GG], forms two monomeric four-layer antiparallel G-quadruplexes which differ in the donor-acceptor directionalities for each individual hydrogen-bonded pair in the G-quartets at pH5.8 and 7.2 respectively.

Table S2. Crystallographic data collection and refinement statistics

**Data collection**	d(G4C2)$_2$-Ba (C222$_1$)	d(G4C2)$_2$-Ba (F222)	d(G4C2)$_2$-K
Space group	C222$_1$	F222	F222
Wavelength (Å)	0.97890	0. 97913	0.97911
Unit cell parameters (Å)	a=60.841<br>b=110.076<br>c=56.675<br>α=β=γ=90°	a=55.613<br>b=60.817<br>c=109.605<br>α=β=γ=90°	a=57.216<br>b=61.059<br>c=110.427<br>α=β=γ=90°
Resolution range (Å)	50-1.60 (1.63-1.60)	50-1.97 (2.00-1.97)	50-2.38 (2.42-2.38)
No. of unique reflections	25435 (1165)	6636 (301)	3951 (192)
Redundancy	12.0 (9.9)	10.5 (6.5)	10.4 (8.4)
I/σ	51.45 (2.74)	61.1 (2.2)	48.6 (4.2)
Completeness (%)	99.3 (92.8)	97.9 (93.2)	97.8 (91.4)
R_{merge}[a] (%)	8.6 (68.6)	8.3 (87.0)	8.4 (45.5)
Anomalous completeness (%)	99.1 (91.2)	97.7 (90.8)	-
Anomalous multiplicity	6.3 (5.2)	5.5 (3.0)	-
**Structure refinement**			
Resolution (Å)	1.60	1.97	2.38
R_{work}[b] (%)	14.09	23.57	21.50
R_{free}[c] (%)	17.12	25.37	25.98
RMSD bonds (Å)	0.012	0.007	0.008
RMSD angles (°)	1.547	1.286	1.429
Average B factor (Å^2)	48.6	65.6	72.3
No. of atoms			
DNA	1494	690	766
Water	82	11	6
ion	7	5	8

Numbers in parentheses represent the values for the highest-resolution shell.

[a]$R_{merge} = \Sigma|I_i - \langle I\rangle|/\Sigma I_i$, where I_i is the intensity of measured reflection and $\langle I\rangle$ is the mean intensity of all symmetry-related reflections.

[b]$R_{work} = \Sigma_W||F_{calc}| - |F_{obs}||/\Sigma|F_{obs}|$, where F_{obs} and F_{calc} are observed and calculated structure factors. W is working dataset of about 95% of the total unique reflections randomly chosen and used for refinement.

[c]$R_{free} = \Sigma_T||F_{calc}| - |F_{obs}||/\Sigma|F_{obs}|$, where T is a test dataset of about 5% of the total unique reflections randomly chosen and set aside prior to refinement.

Figure S1. (A) The initial SAD map of d(G4C2)$_2$-Ba in C222$_1$ space group contoured at 1.0 σ. (B) The anomalous difference map of d(G4C2)$_2$-Ba in C222$_1$ space group contoured at 3.0 σ and the positions of Ba^{2+} atoms. (C) A list of orthogonal coordinates of Ba^{2+} atoms and the height of the related peaks in anomalous difference map of d(G4C2)$_2$-Ba (C222$_1$).

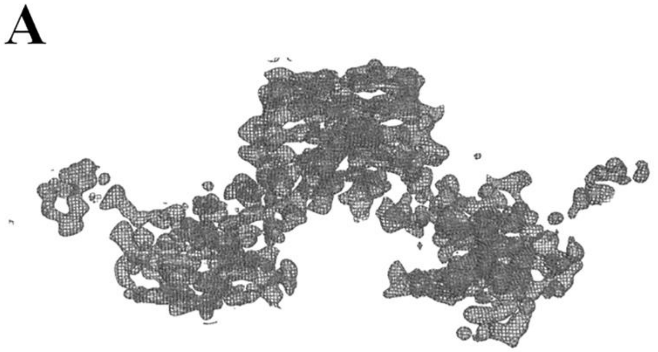

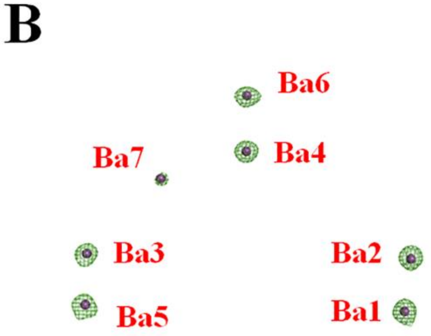

C

Orders	Orthogonal coordinates (Å)			Height (σ)	Ba index
1	27.55	101.19	43.76	28.36	Ba1
2	21.66	101.07	46.58	27.79	Ba2
3	21.48	65.59	38.96	23.98	Ba3
4	9.62	83.27	42.13	23.81	Ba4
5	27.29	65.48	41.54	20.53	Ba5
6	3.33	83.29	42.32	19.81	Ba6
7	9.66	82.56	42.07	13.94	close to Ba4
8	3.30	82.56	42.29	11.77	close to Ba6
9	13.50	73.52	45.03	5.51	Ba7

Figure S2. (A) The initial SAD map of d(G4C2)$_2$-Ba in F222 space group contoured at 1σ. (B) The anomalous difference map of d(G4C2)$_2$-Ba in F222 space group contoured at 3σ and the positions of Ba^{2+} atoms. (C) A list of orthogonal coordinates of Ba^{2+} atoms and the height of the related peaks in anomalous difference map of d(G4C2)$_2$-Ba (F222).

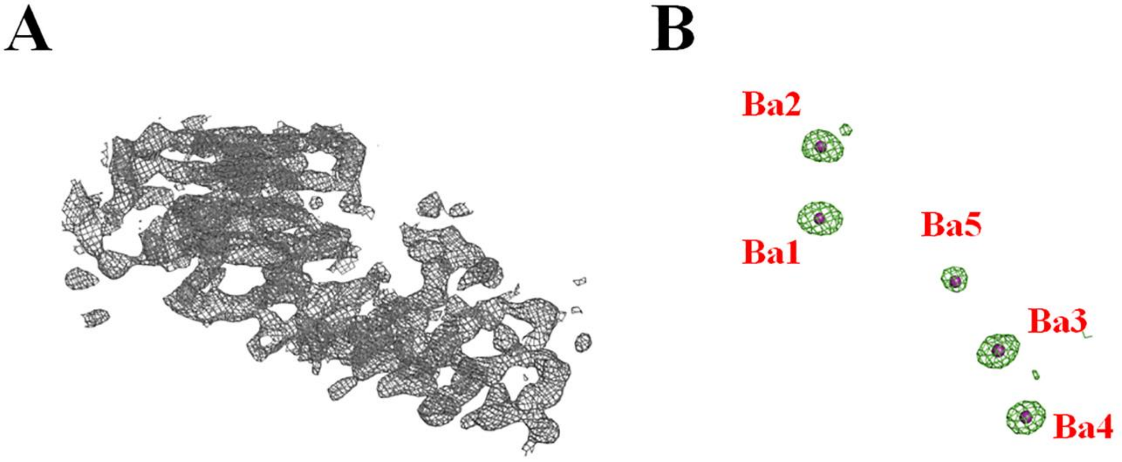

C

**Orders**	**Orthogonal coordinates (Å)**			**Height (σ)**	**Ba index**
**1**	-13.90	12.02	-27.40	24.5	Ba4
**2**	-13.90	5.56	-27.40	19.85	Ba3
**3**	-15.00	-12.24	-9.62	18.40	Ba2
**4**	-17.82	-6.40	-9.74	18.18	Ba1
**5**	-19.90	-11.98	-9.89	8.08	close to Ba3
**6**	-11.57	1.70	-18.32	5.51	Ba5

Figure S3. The crystal structure of d(G4C2)$_2$-Ba in C222$_1$ space group. (A) An asymmetric unit of d(G4C2)$_2$ in Ba^{2+} (purple spheres) contains 6 chains, A-F. The observed non-central channel Ba^{2+} shown by arrow. (B) Crystal packing of d(G4C2)$_2$-Ba (C222$_1$). The Form-1/7 formed by chains A/B and Form-1/1 formed by chains C/D or chains E/F are indicated by dashed rectangles.

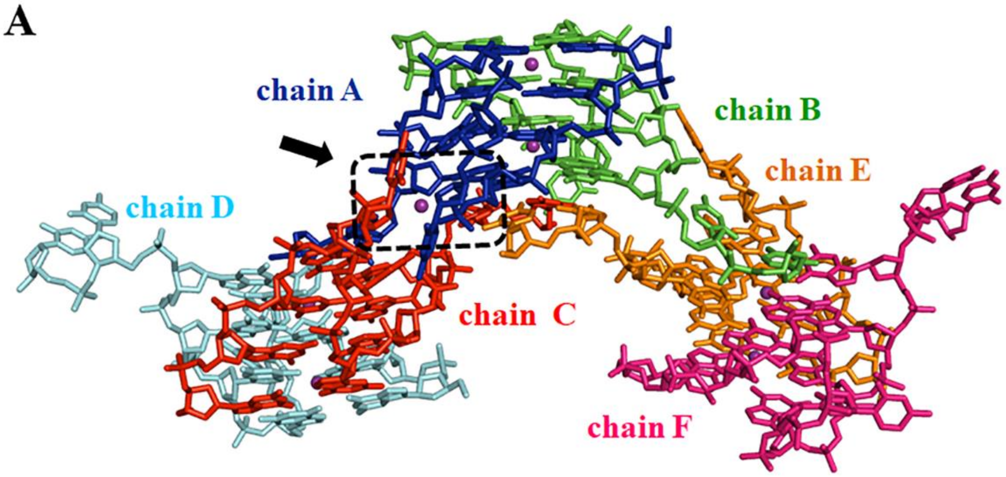

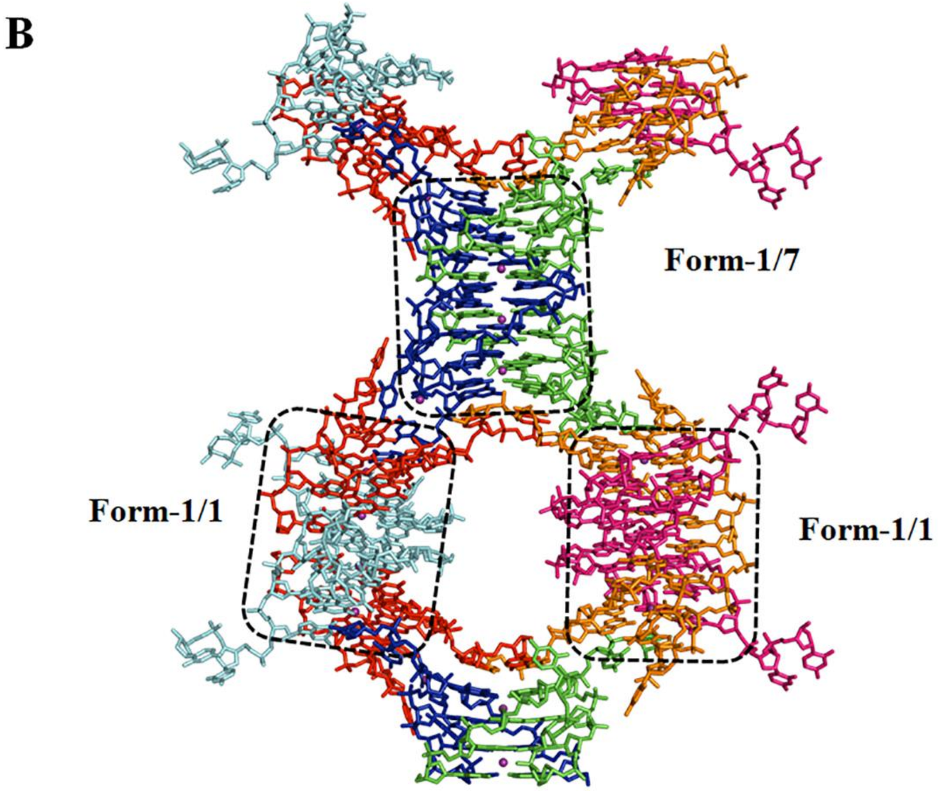

Figure S4. The G-tetrad base stacking mode at the interface of two dimeric block in the tetrameric G-quadruplex crystal structure of d(G4C2)$_2$-Ba (C222$_1$). (A) and (B) represent Form-1/7 and Form-1/1 with different colors for each of the four strands, respectively. The hydrogen bonds are represented by solid black lines.

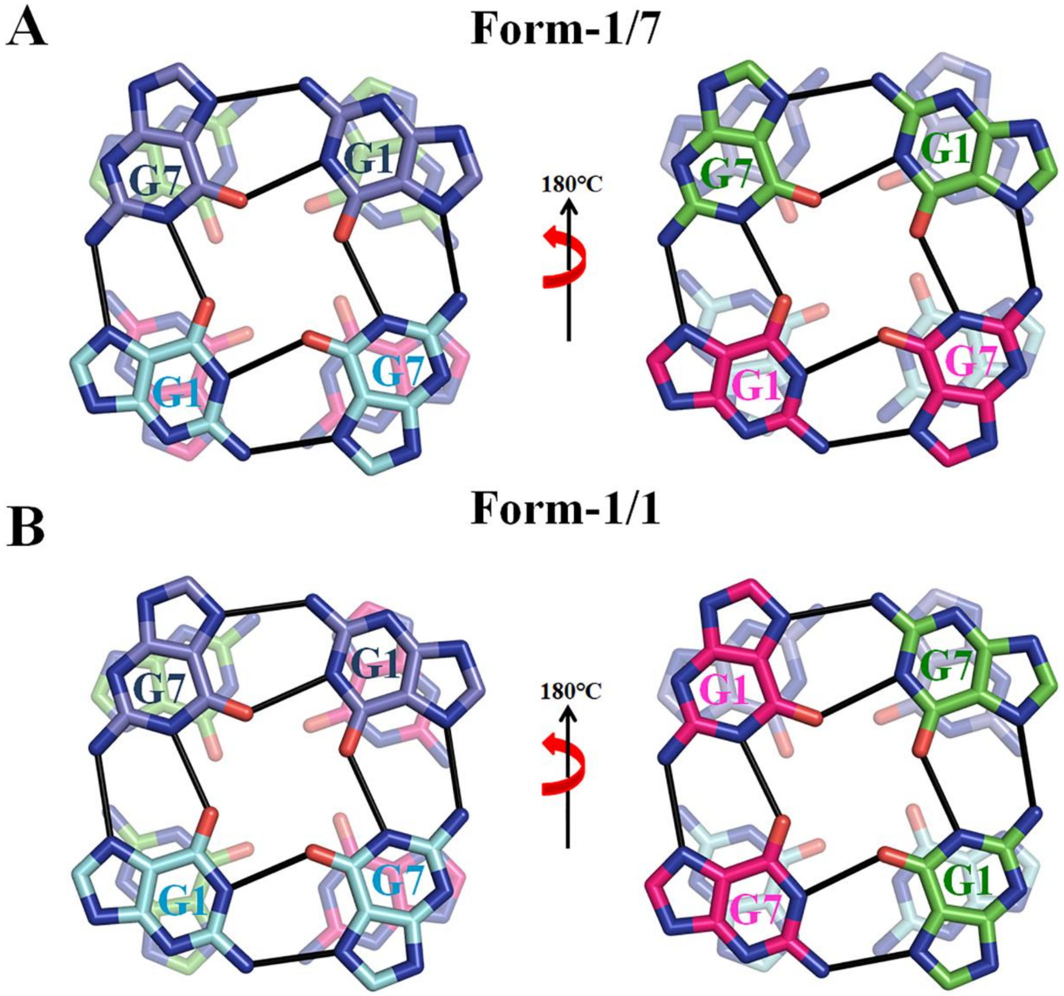

Figure S5. Left: The electron density corresponds to the final 2mFo-DFc map contoured at 1.0 σ for d(G4C2)$_2$-Ba (C222$_1$). Right: Expanded view of the environment of the Ba^{2+} ions in the tetrameric G-quadruplex formed by d(G4C2)$_2$, with the bonds between Ba^{2+} and oxygen atom shown as solid black lines. (A) and (B) represent Form-1/7 and Form-1/1 with carbon atoms colored different for each of the four strands, respectively.

A **Form-1/7**

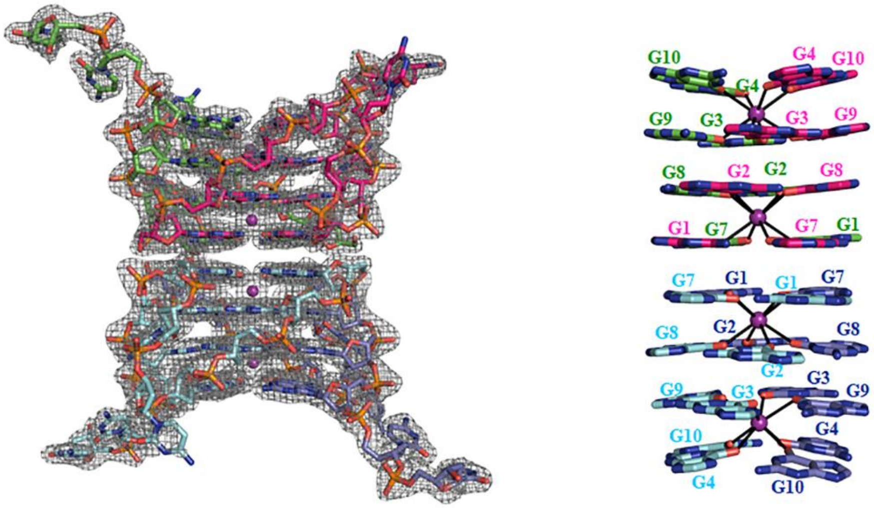

B **Form-1/1**

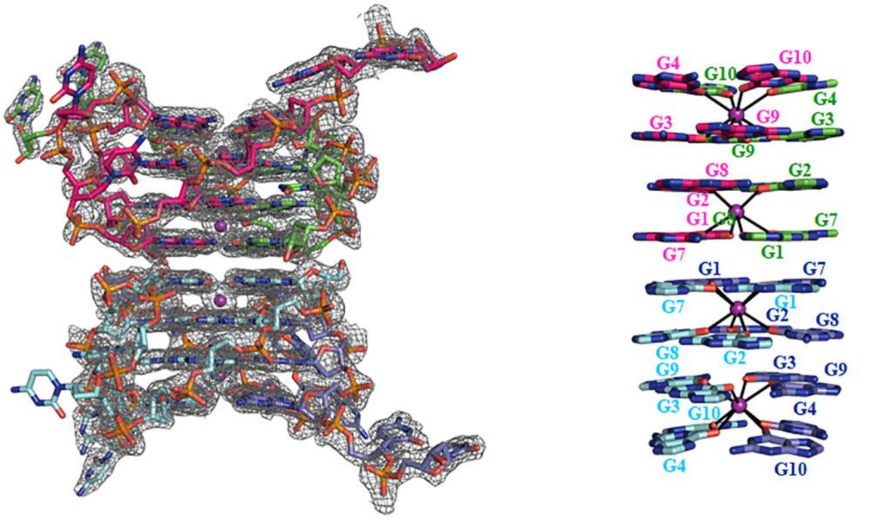

Figure S6. The view of the additional Ba^{2+} ion observed in the crystal structure of $d(G4C2)_2$-Ba ($C222_1$). The hydrogen bonds are represented by solid black lines. Water molecules are colored red and Ba^{2+} atom is in purple sphere.

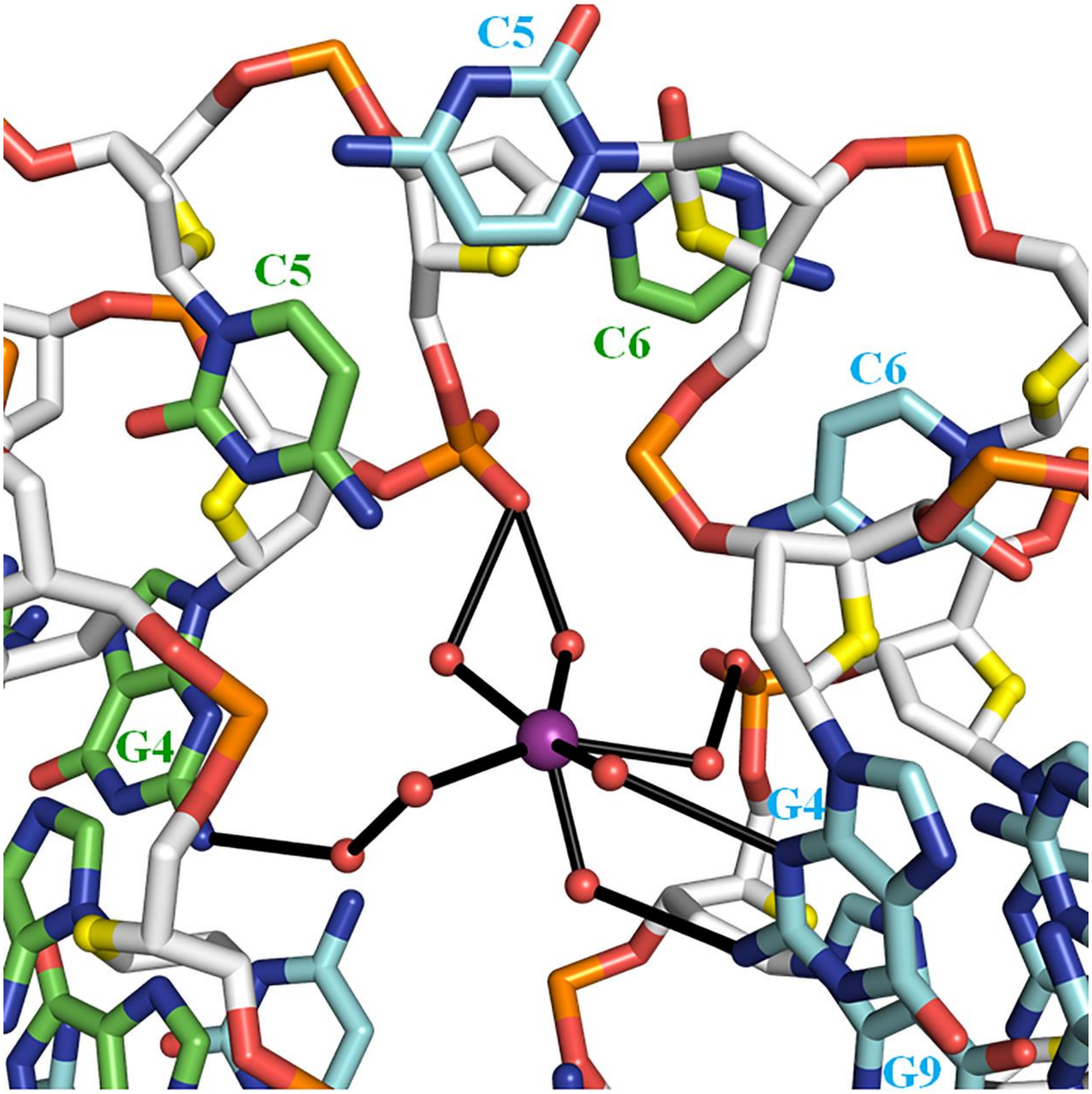

Figure S7. The view of the cytosines in the crystal structure of d(G4C2)$_2$-Ba (C222$_1$). The C5, C6, C11 and C12 bases in (A) the Form-1/7 formed by chains A/B, (B) Form-1/1 formed by chains C/D and (C) Form-1/1 formed by chains E/F. The electron density corresponds to the final 2mFo-DFc map contoured at 1.0 σ. The G-tetrad core of the tetrameric G-quadruplex is shown in surface mode with the atoms of phosphate and phosphate oxygen colored in red.

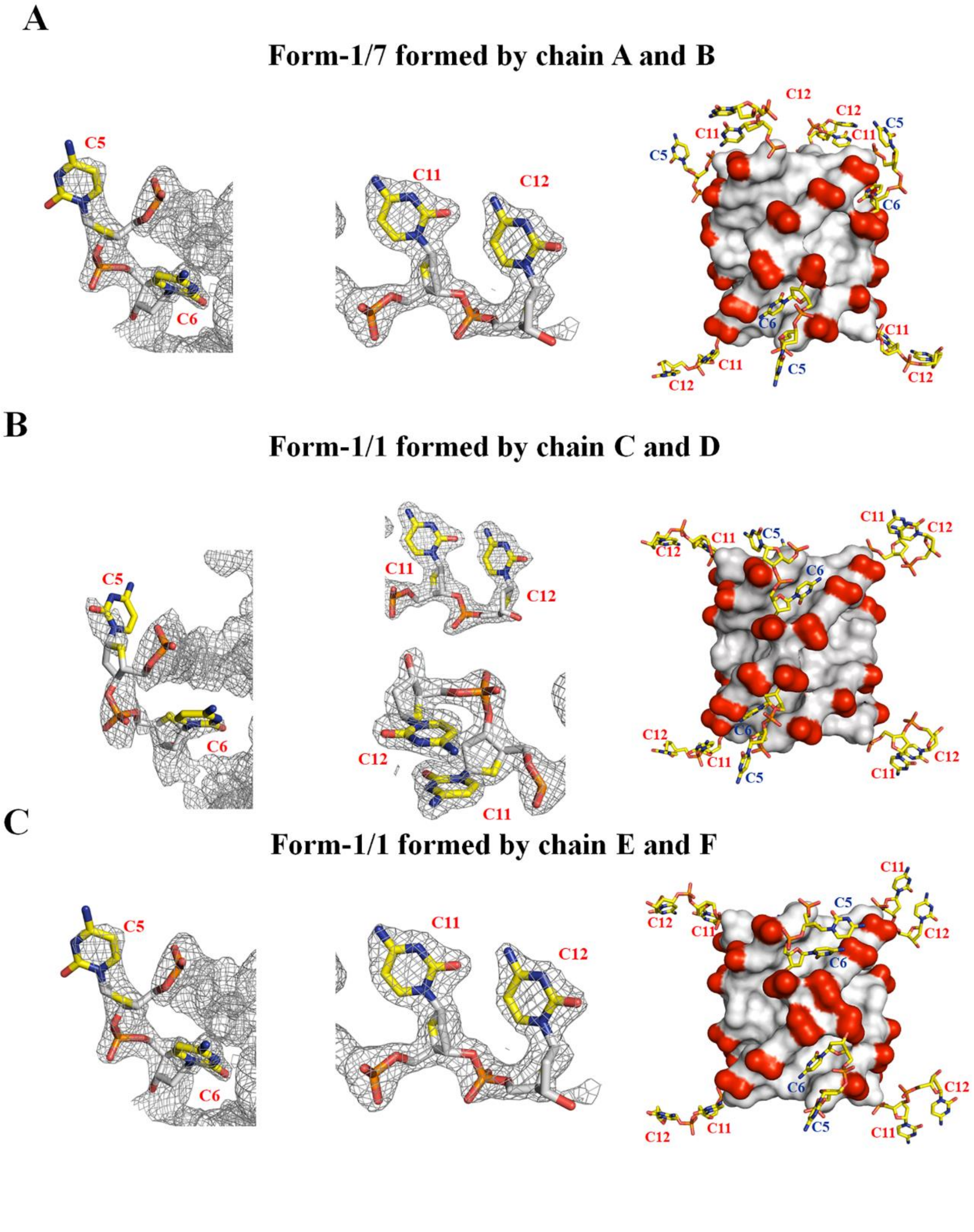

Figure S8. The crystal structure of d(G4C2)$_2$ in K^+ solution in F222 space group. (A) An asymmetric unit of d(G4C2)$_2$ in K^+ (magenta spheres) solution contains 3 chains, A-C. (B) Crystal packing of d(G4C2)$_2$-K. The Form-1/7 and Form-1/1 are indicated by dashed rectangle.

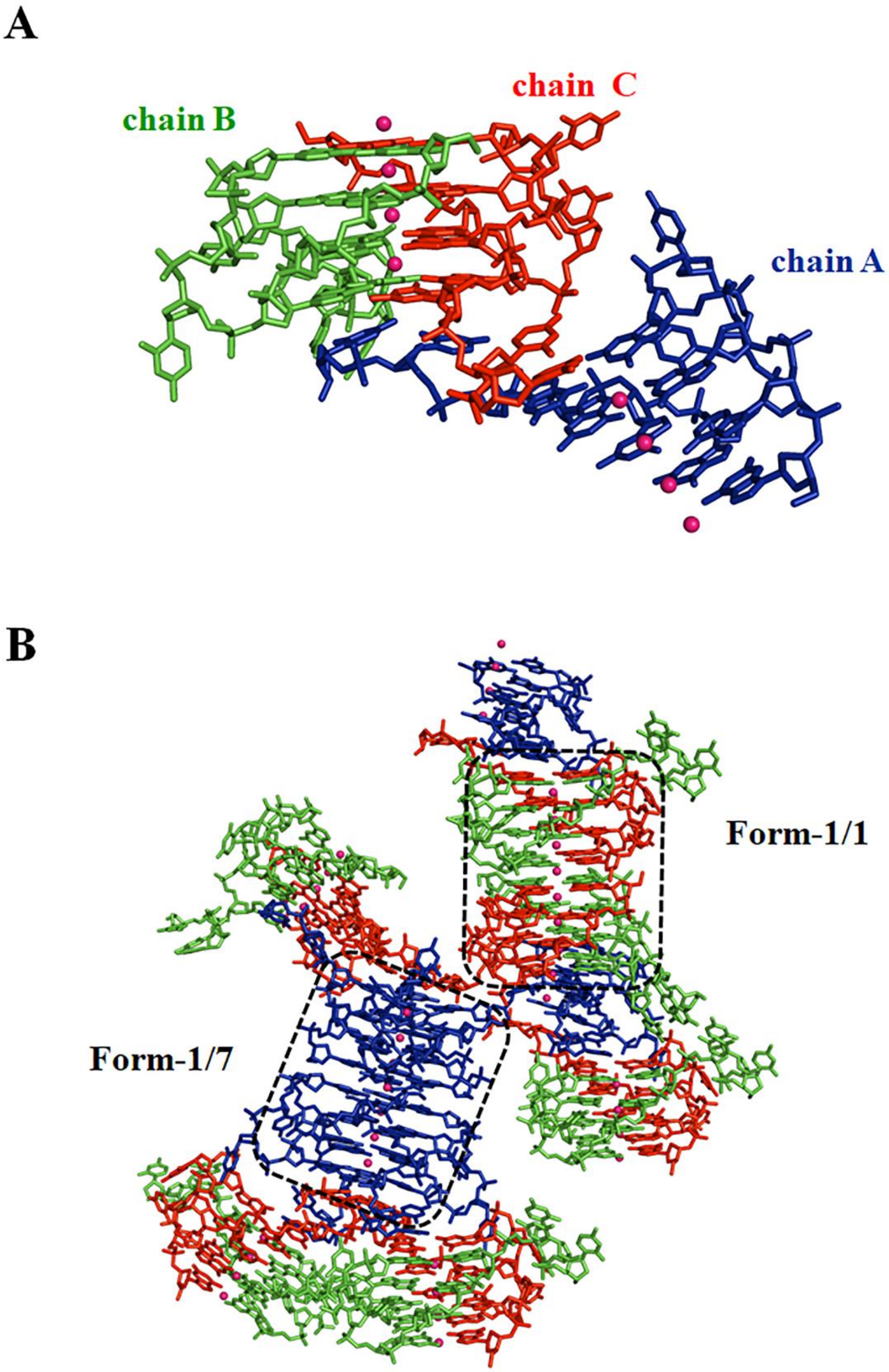

Figure S9. The crystal structure of d(G4C2)$_2$ in Ba^{2+} solution in F222 space group. (A) An asymmetric unit of d(G4C2)$_2$ in Ba^{2+} (purple spheres) solution contains 3 chains, A-C. (B) Crystal packing of d(G4C2)$_2$-Ba (F222). The Form-1/7 and Form-1/1 are indicated by dashed rectangle.

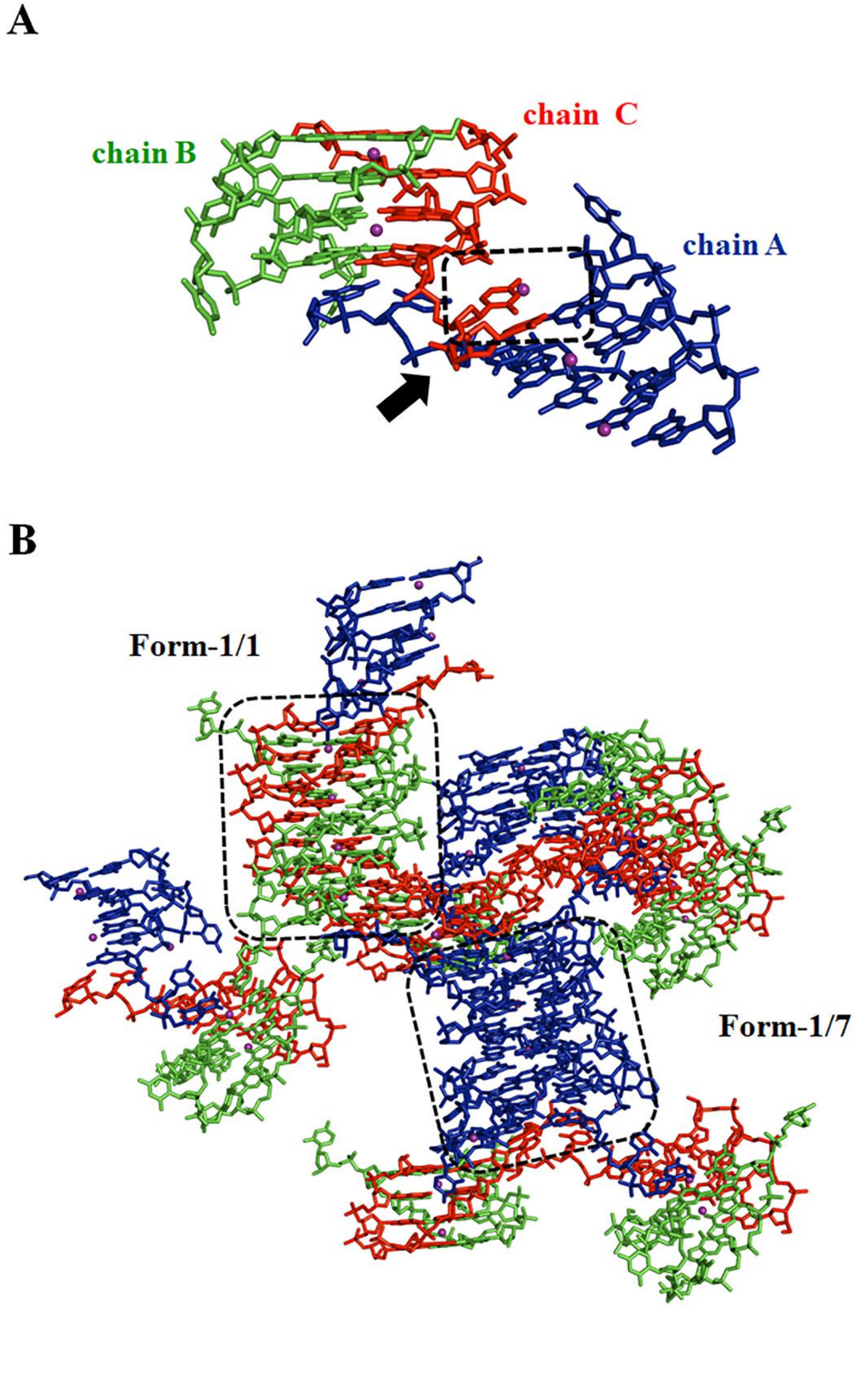

Figure S10. Crystal structure of d(G4C2)$_2$-Ba in F222 space group. (A) Form-1/7 and (B) Form-1/1 in which each dimeric block is stacked to form a tetrameric G-quadruplex via different 5'-arrangments and stabilized by Ba^{2+} (purple sphere). Left: Cartoon representation of tetrameric G-quadruplex formed by d(G4C2)$_2$. Middle: Schematic representation of topology adopted by d(G4C2)$_2$. Right: Top view of Left. Each molecule, d(G4C2)$_2$, is shown as red, green, blue and cyan in the tetrameric G-quadruplex. O4' oxygens are in yellow. Dashes represent the missing residues, the CC loops.

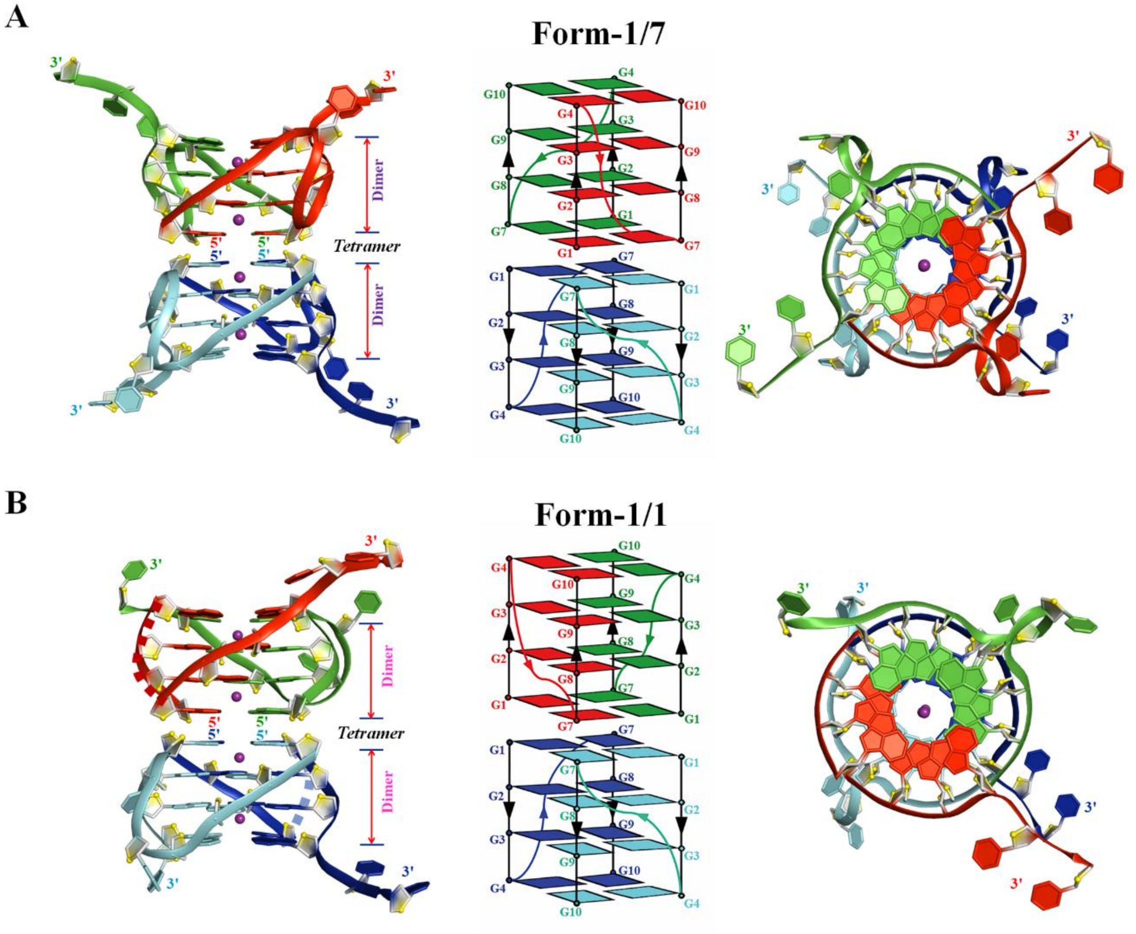

Figure S11. Left: The electron density corresponds to the final 2mFo-DFc map contoured at 1.0 σ for $d(G4C2)_2$-K. Right: Expanded view of the environment of the K^+ ions in the tetrameric G-quadruplex formed by $d(G4C2)_2$, with the bonds between K^+ and oxygen atom shown as solid black lines. (A) and (B) represent Form-1/7 and Form-1/1 with carbon atoms colored different for each of the four strands, respectively.

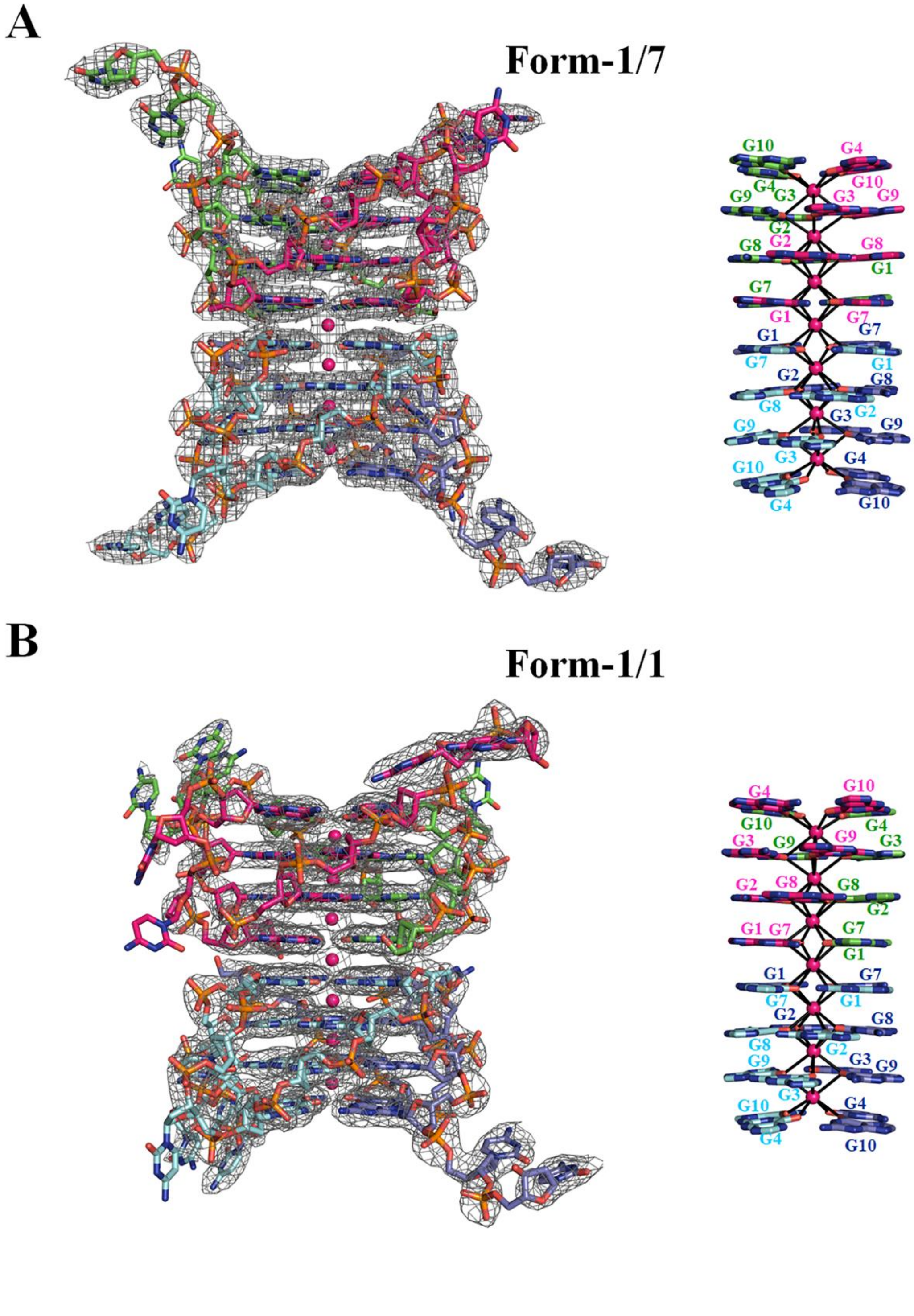

Figure S12. The view of the cytosines in the crystal structure of d(G4C2)$_2$-K. The C5, C6, C11 and C12 bases in (A) the Form-1/7 formed by chain A and its crystallographically symmetric molecules, (B) Form-1/1 formed by chains B/C and their crystallographically symmetric molecules. The electron density corresponds to the final 2mFo-DFc map contoured at 1.0 σ. The G-tetrad core of the tetrameric G-quadruplex is shown in surface mode with the atoms of phosphate and phosphate oxygen colored in red..

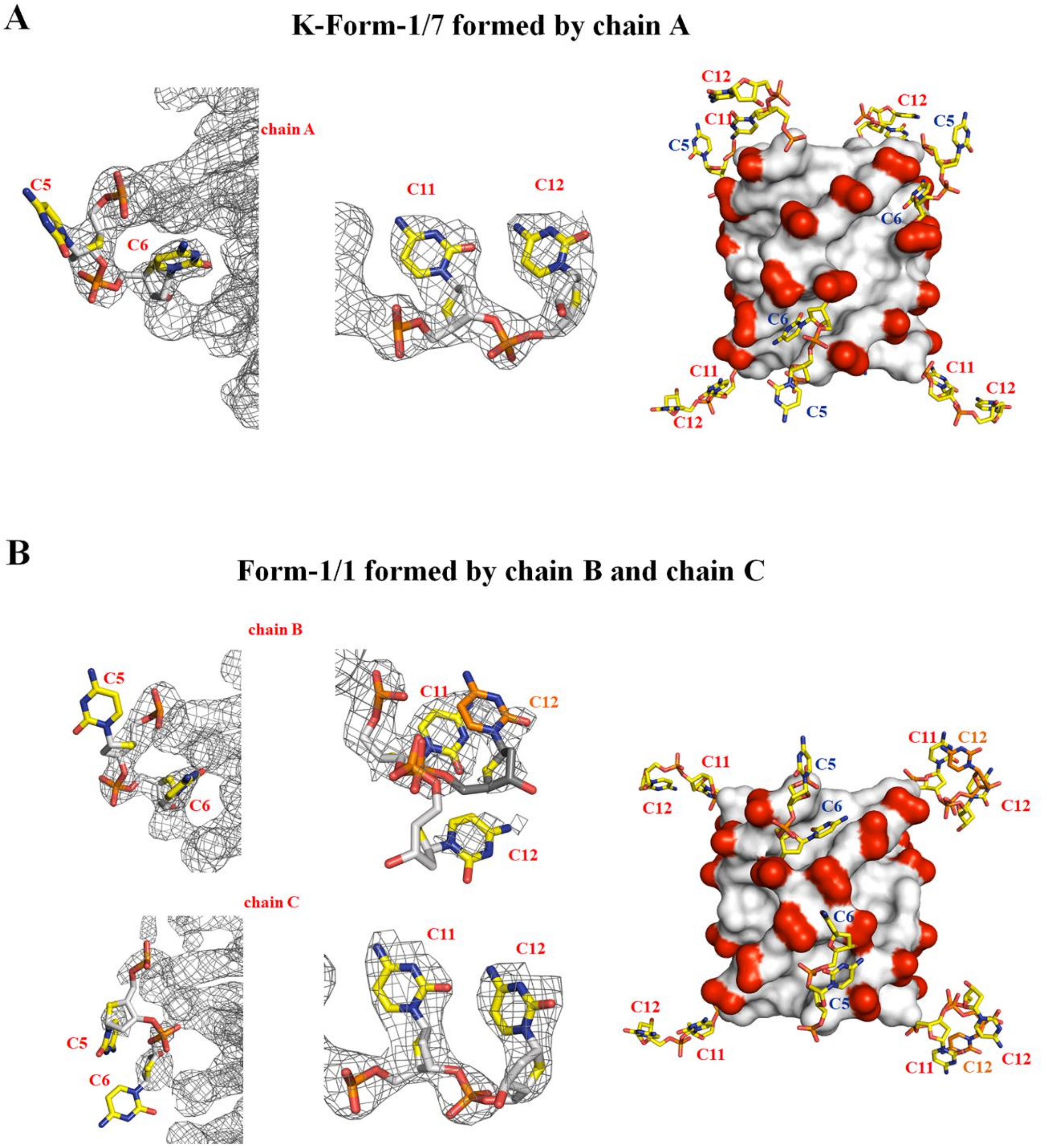

Figure S13. Detailed conformations of cytosines in the structure of d(G4C2)$_2$-Ba (F222). The conformation of propeller loop, C5 and C6, in the dimeric G-quadruplex of (A) Form-1/7 and (B) Form-1/1. (C) The conformation of the C11 and C12 bases of chain C forming Form-1/1 located at the 3'- end. (D) The conformation of the C11 and C12 bases of chain A (yellow) forming Form-1/7 and B (green) forming Form-1/1. The hydrogen bonds are represented by solid black lines.

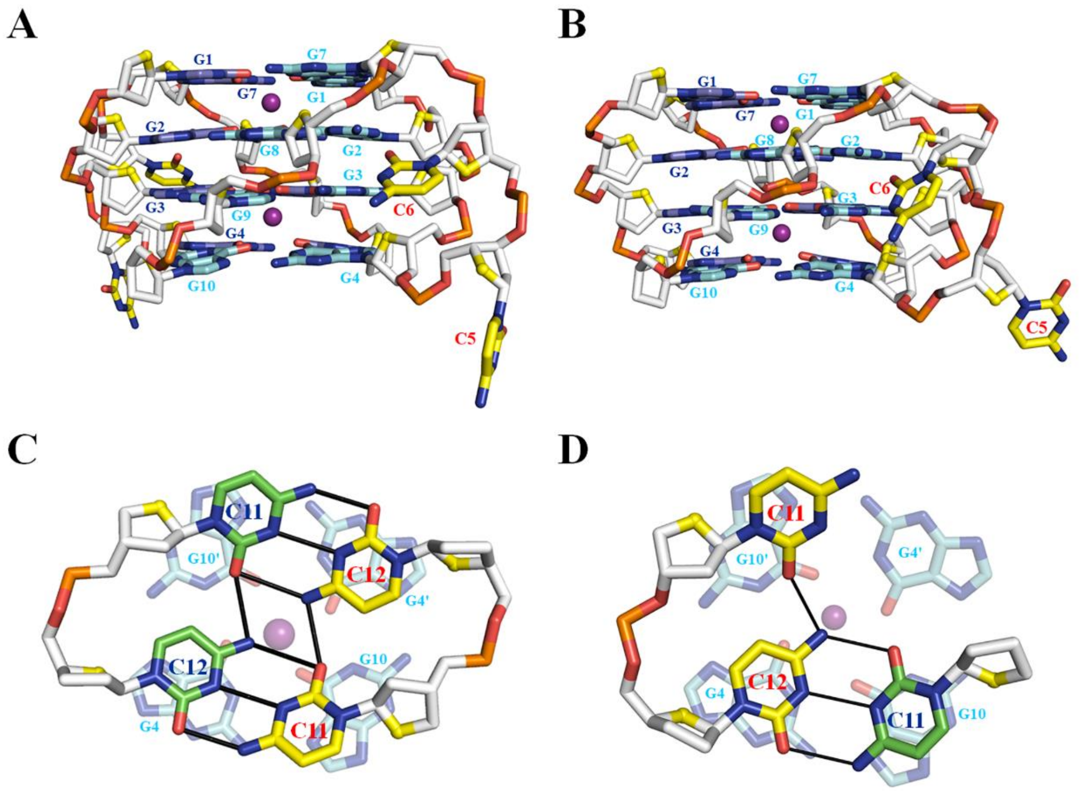

Figure S14. Intermolecular π−π packing interactions for cytosine bases observed in the structure of (A) d(G4C2)$_2$-Ba (F222) Form-1/7 formed by chain A and (B) d(G4C2)$_2$-K Form-1/7 formed by chain A. The prime (′) notation signifies that the two bases belong to separate oligonucleotide strands.

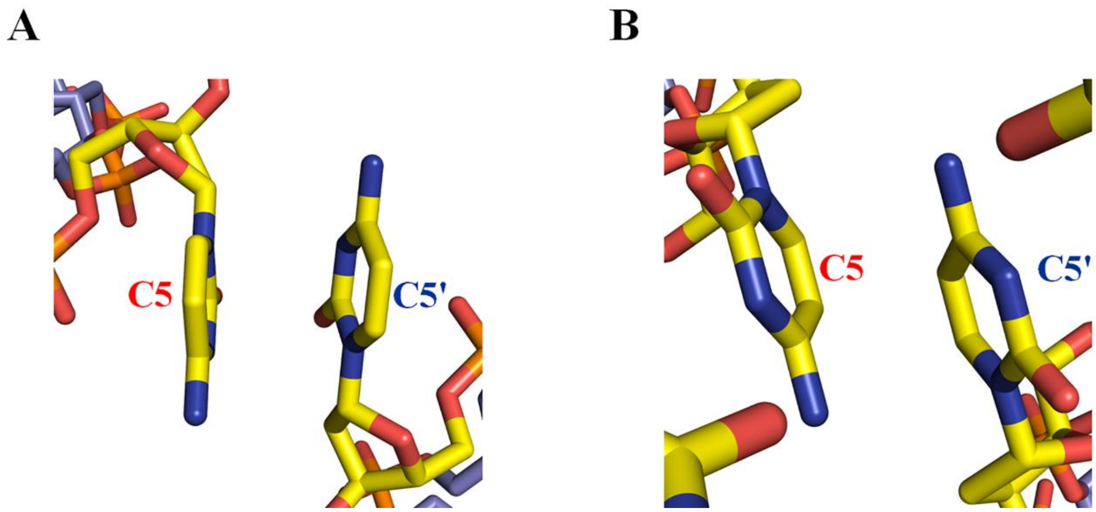

References

1. Zhou, B.; Liu, C.; Geng, Y.; Zhu, G., Topology of a G-quadruplex DNA formed by C9orf72 hexanucleotide repeats associated with ALS and FTD. *Scientific reports* **2015,** *5*, 16673.
2. Zhou, B.; Geng, Y. Y.; Liu, C. D.; Miao, H. T.; Ren, Y. G.; Xu, N. N.; Shi, X.; You, Y. Y.; Lee, T. L.; Zhu, G., Characterizations of distinct parallel and antiparallel G-quadruplexes formed by two-repeat ALS and FTD related GGGGCC sequence. *Scientific reports* **2018,** *8*.
3. Sket, P.; Pohleven, J.; Kovanda, A.; Stalekar, M.; Zupunski, V.; Zalar, M.; Plavec, J.; Rogelj, B., Characterization of DNA G-quadruplex species forming from C9ORF72 G4C2-expanded repeats associated with amyotrophic lateral sclerosis and frontotemporal lobar degeneration. *Neurobiol Aging* **2015,** *36* (2), 1091-6.
4. Brcic, J.; Plavec, J., Solution structure of a DNA quadruplex containing ALS and FTD related GGGGCC repeat stabilized by 8-bromodeoxyguanosine substitution. *Nucleic Acids Res* **2015,** *43* (17), 8590-8600.
5. Brcic, J.; Plavec, J., NMR structure of a G-quadruplex formed by four d(G4C2) repeats: insights into structural polymorphism. *Nucleic Acids Res* **2018,** *46* (21), 11605-11617.
